# Effect of Financial Incentives and Environmental Strategies on Weight Loss in the Healthy Weigh Study: A Randomized Clinical Trial

**DOI:** 10.1001/jamanetworkopen.2021.24132

**Published:** 2021-09-07

**Authors:** Karen Glanz, Pamela A. Shaw, Pui L. Kwong, Ji Rebekah Choi, Annie Chung, Jingsan Zhu, Qian Erin Huang, Karen Hoffer, Kevin G. Volpp

**Affiliations:** 1 Department of Biostatistics, Epidemiology, and Informatics, Perelman School of Medicine, University of Pennsylvania, Philadelphia; 2 School of Nursing, University of Pennsylvania, Philadelphia; 3 Department of Medical Ethics and Health Policy, Perelman School of Medicine, University of Pennsylvania, Philadelphia; 4 Children’s Hospital of Philadelphia, Philadelphia, Pennsylvania; 5 Department of Medicine, Perelman School of Medicine, University of Pennsylvania, Philadelphia

## Abstract

**Importance:**

Modest weight loss can lead to meaningful risk reduction in adults with obesity. Although both behavioral economic incentives and environmental change strategies have shown promise for initial weight loss, to date they have not been combined, or compared, in a randomized clinical trial.

**Objective:**

To test the relative effectiveness of financial incentives and environmental strategies, alone and in combination, on initial weight loss and maintenance of weight loss in adults with obesity.

**Design, Setting, and Participants:**

This randomized clinical trial was conducted from 2015 to 2019 at 3 large employers in Philadelphia, Pennsylvania. A 2-by-2 factorial design was used to compare the effects of lottery-based financial incentives, environmental strategies, and their combination vs usual care on weight loss and maintenance. Interventions were delivered via website, text messages, and social media. Participants included adult employees with a body mass index (BMI; weight in kilograms divided by height in meters squared) of 30 to 55 and at least 1 other cardiovascular risk factor. Data analysis was performed from June to July 2021.

**Interventions:**

Interventions included lottery-based financial incentives based on meeting weight loss goals, environmental change strategies tailored for individuals and delivered by text messages and social media, and combined incentives and environmental strategies.

**Main Outcome and Measures:**

The primary outcome was weight change from baseline to 18 months, measured in person.

**Results:**

A total of 344 participants were enrolled, with 86 participants each randomized to the financial incentives group, environmental strategies group, combined financial incentives and environmental strategies group, and usual care (control) group. Participants had a mean (SD) age of 45.6 (10.5) years and a mean (SD) BMI of 36.5 (7.1); 247 participants (71.8%) were women, 172 (50.0%) were Black, and 138 (40.1%) were White. At the primary end point of 18 months, participants in the incentives group lost a mean of 5.4 lb (95% CI, −11.3 to 0.5 lb [mean, 2.45 kg; 95% CI, −5.09 to 0.23 kg]), those in the environmental strategies group lost a mean of a 2.2 lb (95% CI, −7.7 to 3.3 lb [mean, 1.00 kg; 95% CI, −3.47 to 1.49 kg]), and the combination group lost a mean of 2.4 lb (95% CI, −8.2 to 3.3 lb [mean, 1.09 kg; 95% CI, −3.69 to 1.49 kg]) more than participants in the usual care group. Financial incentives, environmental change strategies, and their combination were not significantly more effective than usual care. At 24 months, after 6 months without an intervention, the difference in the change from baseline was similar to the 18-month results, with no significant differences among groups.

**Conclusions and Relevance:**

In this randomized clinical trial, across all study groups, participants lost a modest amount of weight but those who received financial incentives, environmental change, or the combined intervention did not lose significantly more weight than those in the usual care group. Employees with obesity may benefit from more intensive individualized weight loss strategies.

**Trial Registration:**

ClinicalTrials.gov Identifier: NCT02878343

## Key Points

**Question:**

What is the relative effectiveness of financial incentives and environmental change strategies, alone and in combination, on initial weight loss and maintenance of weight loss in employees with obesity?

**Findings:**

In this randomized clinical trial, at the 18-month primary end point, participants in the incentives group lost a mean of 5.4 lb (2.45 kg), those in the environmental strategies group lost a mean of 2.2 lb (1.00 kg), and the combination group lost a mean of 2.4 lb (1.09 kg) more than the usual care group. None of the strategies was significantly more effective than usual care.

**Meaning:**

Across all study groups, participants lost a modest amount of weight but those who received interventions did not lose significantly more weight, suggesting that more intensive individualized weight loss strategies may be needed.

## Teaser

This randomized clinical trial tests the relative effectiveness of financial incentives and environmental strategies, alone and in combination, on initial weight loss and maintenance of weight loss in employees with obesity at 3 large employers in Philadelphia, Pennsylvania.

## Introduction

The prevalence of obesity has increased significantly in the US in recent decades. Between 1960 and 2014, the prevalence of obesity—defined as a body mass index (BMI; weight in kilograms divided by height in meters squared) of more than 30—among US adults increased from 11.0% to 35.0% among men and from 16.0% to 40.4% among women.^1^ Obesity in adulthood is associated with higher rates of cardiovascular risk factors, disability, hospitalization, health care expenditures, and mortality risk.^2,3,4^ A modest amount of weight loss, approximately 10 lb (4.5 kg), can reduce the incidence of diabetes and improve risk factors such as hypertension and hyperglycemia.^5,6^ A number of approaches are successful in achieving initial weight loss,^7^ but maintaining weight loss is challenging.^8,9^

Although both behavioral economic and environmental strategies have shown promise, to date they have not been combined, or compared, in a randomized clinical trial. Financial incentives have been shown to modify health behaviors,^10,11,12,13,14^ including inducing initial weight loss.^12,13,14,15^ Environmental change strategies, such as providing easily accessible healthful foods and building exercise opportunities into workplace design, have been proposed as critical to solving the population-wide problem of obesity^16^ and have been shown to affect food intake and physical activity.^16,17,18,19,20,21^ Some environmental intervention strategies in workplaces have helped to increase physical activity and promote weight control.^21^ However, their effectiveness has not been tested in the context of long-term weight loss or weight maintenance or when combined with financial incentives. Furthermore, the comparative effectiveness of incentive and environmental strategies is not known.^22^ We, therefore, undertook the present study to answer the following question: How effective are financial incentives and environmental strategies, alone and in combination, on initial weight loss and maintenance of weight loss (primary outcome at 18 months; secondary outcome at 24 months) in employed adults with obesity?

## Methods

### Overview of Study Design

The Healthy Weigh Study is a randomized clinical trial evaluating the comparative effectiveness of lottery-based incentive and environmental strategies, alone or in combination, to a control intervention in a population of urban employees with obesity.^22^ The study was conducted from 2015 to 2019. Details of the protocol and baseline findings have been reported elsewhere,^22^ and the protocol is shown in Supplement 1. The study had a 2-by-2 factorial design. Participants were randomized in a 1:1:1:1 ratio to receive 1 of 4 interventions for 18 months: (1) a lottery-based financial incentive, (2) environmental strategies, (3) both the lottery incentive and environmental strategies, or (4) a usual care (control) intervention with standard employee wellness benefits and weigh-ins every 6 months. All groups received the usual care intervention. After randomization, participants were aware of the intervention they received. In the first 6 months, active interventions focused on weight loss, and in months 7 to 18, the focus was on maintenance or continued weight loss. After the primary end point of 18 months, participants were followed up for an additional 6 months without any intervention. This study was approved by the institutional review board of the University of Pennsylvania, and participants completed informed consent forms online. This randomized clinical trial follows the Consolidated Standards of Reporting Trials (CONSORT) reporting guideline.

### Study Population

Eligible participants were men and women who were aged at least 18 years, working full-time or part-time at 3 large employers in Philadelphia, Pennsylvania, and who had a BMI of 30 to 55 and at least 1 other cardiovascular risk factor. At the time of recruitment, participants also must have as their primary health insurance provider Independence Blue Cross, one of the companies participating in the study. Background information, including race and ethnicity, was collected by self-report to characterize participants’ demographic characteristics. Individuals who could not read consent forms or complete surveys in English were excluded. Health-related exclusion criteria were limited to factors that make participation in a weight loss program unfeasible, unsafe, or require more intensive monitoring or that may confound results,^23,24^ such as unstable heart disease, serious chronic illness (eg, transplant recipient, terminal illness), substance abuse, or pregnancy.

### Recruitment

For this study, we collaborated with 3 large urban employers in Philadelphia, Pennsylvania. Initial outreach to employees used existing communication channels at each site.^22^ Interested participants were asked to access the Penn Way to Health (WTH) study platform for prescreening and eligibility ascertainment. WTH is a customizable web-based platform that supports recruitment, consent, randomization, and data collection for clinical trials.^25^

### Interventions

All interventions were conducted for 18 months. Participants in the 3 intervention groups received a free wireless scale (accurate to within 0.2 lb [0.09 kg]) to submit weights remotely. Participants in the usual care group did not receive wireless scales because it would not be consistent with usual care.

#### Financial Incentives

The lottery-based incentive group participants were given a weight-loss goal for the first 6 months of 0.5 lb (0.23 kg) a week. For each month from months 7 to 18, they were asked to set a goal to either maintain weight loss or lose more weight. Participants weighed themselves each morning, and their weights were automatically transmitted to the WTH system. Participants received automated verbal and graphic feedback on their progress relative to their goals and potential earnings.

Participants in the lottery group were eligible for a daily lottery prize with an expected value of $3.00 per day if they met their weight goal. Half of the winnings were paid out at the end of each month. To leverage loss aversion and the endowment effect to augment motivation,^26^ the other half was held in a virtual account and paid after 6 months if participants met or exceeded their monthly goals.

#### Environmental Strategies

The environmental change strategy group involved a menu of promising and evidence-based environmental change strategies to promote healthy eating and physical activity, delivered through mobile and website-based communication channels to employees in the environmental and combined groups of the trial. The interventions are targeted at individual employees rather than work groups. Healthy eating environmental change strategies guided participants in identifying environmental influences on excess food intake and making environmental modifications in or near their workplaces and in their homes. For example, workplace-based strategies emphasized identifying healthful vending machine options and healthful snack access. Home environment changes were guided by the Home Food and Activity Environment Audit tool.^27,28^ Physical activity environment change strategies also included strategies for workplaces, near workplaces, and in homes.^27^

Environmental change strategies (tips and messages) were sent to participants in the environmental and combined study groups twice weekly during the first 6 months, and weekly during months 7 to 18. Participants in the environmental strategies groups were also invited to join a private, members-only Facebook group, where group members were invited to share their stories to motivate each other with their weight loss.

#### Usual Care

Employees at all 3 workplaces were offered a wellness program that consisted of yearly biometric screenings and reimbursements for fitness and weight management program participation (up to $300 per year). Up to 6 visits with a registered dietitian were also included.

### Outcome Measures

All participants were weighed in person at enrollment into the study and at the visits at 6, 12, 18, and 24 months. The primary outcome was weight loss at 18 months, and the secondary outcome was weight loss or maintenance at 24 months. Participants completed surveys at baseline and every 6 months, to assess background characteristics and potential covariates and mechanisms of change, including physical activity,^29^ cognitive restraint, uncontrolled eating and emotional eating behaviors,^30^ stages of change for diet,^31^ and general health-related quality of life.^32^

### Process Measures

Several process measures were used to help interpret the study findings. These included compliance with home weighing in the 3 study groups that had wireless scales, reactions to environmental strategies (ie, text or web surveys), and participation in the Facebook group in the environmental strategy study groups. An end-of-study survey was administered to all participants at the end of their participation.

### Statistical Analysis

Participants were randomized in a 1:1:1:1 ratio to 1 of the 4 groups using stratified randomization and a permuted block algorithm with variable block size using the WTH software. Randomization was stratified by workplace, sex, and BMI (30-37.9 vs 38-55). The following prespecified pairwise comparisons were tested at 18 months: (1) incentive vs control, (2) environmental strategies vs control, (3) incentive plus environmental strategies vs control, (4) incentive vs incentive plus environmental strategies, and (5) environmental strategies vs incentive plus environmental strategies. Assuming weight losses of 17.6 lb (8.0 kg) in the combined group, 11.0 (5.0 kg) in the single-intervention groups, and 0 lb (0 kg), in the control group and an 11.0 lb (5.0 kg) SD for change in weight at 18 months, 65 participants per group had more than 90% power for each single intervention comparison to control and 87% power for each single comparison with the combined intervention, using the Holm-Bonferroni–corrected level for significance.^33^ A 20% dropout rate was assumed to determine the final planned total sample size of 328 participants. During recruitment, the sample size was increased to 344 because some individuals were unable to set up scales.^22^ As a result of a programming error, the 24 participants enrolled after the change in enrollment targets were randomized using a modified simple randomization algorithm that forced equal size groups of 86 participants by closing groups once the target was achieved.

The primary analysis is an intention-to-treat (ITT) analysis comparing the unadjusted mean weight change from baseline to 18 months (in-person weight), between each intervention group and the control group, and the combined group compared with each single intervention group, using a *t* test and performed at the Holm significance level that adjusts for the 5 primary comparisons.^33^ Multiple imputation using chained equations was used to address missing data, using the randomization strata (sex, employer, or initial BMI), study group and baseline variables (age, race, income, education, marital status, household size, physical activities, eating behavior index, stages of change, 36-Item Short-Form Survey [SF-36] General Health [score range, 1-100, with higher scores denoting better health conditions], and baseline weight) as variables in the imputation model for the 18-month weight outcome. As sensitivity analyses, we also conducted an analysis adjusted for stratification variables (sex, employer, and initial BMI), age, race, income, and education. Other potential confounders were baseline weight, marital status, household size, physical activity (minutes per week), eating behavior index, stages of change, and SF-36 General Health score) which were included in the analysis if indicated by the change in estimate criterion (10%). Similar analyses were performed for 24-month weight outcomes and exploratory end points of total physical activity, cognitive restraint, uncontrolled eating, emotional eating, and SF-36 General Health score.

A per-protocol analysis examined whether the results would be different when including only the more engaged participants, including individuals from all 4 groups who attended their visits at 6, 12, and 18 months. To be included, participants in the environmental strategies group had to use 10 tips or more, incentive group participants had to weigh in an average of at least once a week, and the combined intervention group had to do both.

At-home weekly weigh-in frequency (days per week) was compared among the 3 intervention groups using a generalized estimating equation model, assuming a working independence, using a separate model for the intervention and nonintervention periods. A model with intervention group only was used to estimate the average weighing frequency for each period, and a model with time and a group-time treatment interaction was used to examine whether changes in weighing frequency differed between the intervention groups.

All hypotheses were analyzed using 2-sided tests. The 5 primary comparisons were tested using the Holm-Bonferroni–adjusted significance level, and all other tests were done at the *P* < .05 level. Analyses were conducted using SAS statistical software version 9.4 (SAS Institute, Cary, NC). Data analysis was performed from June to July 2021.

## Results

**Figure 1.**
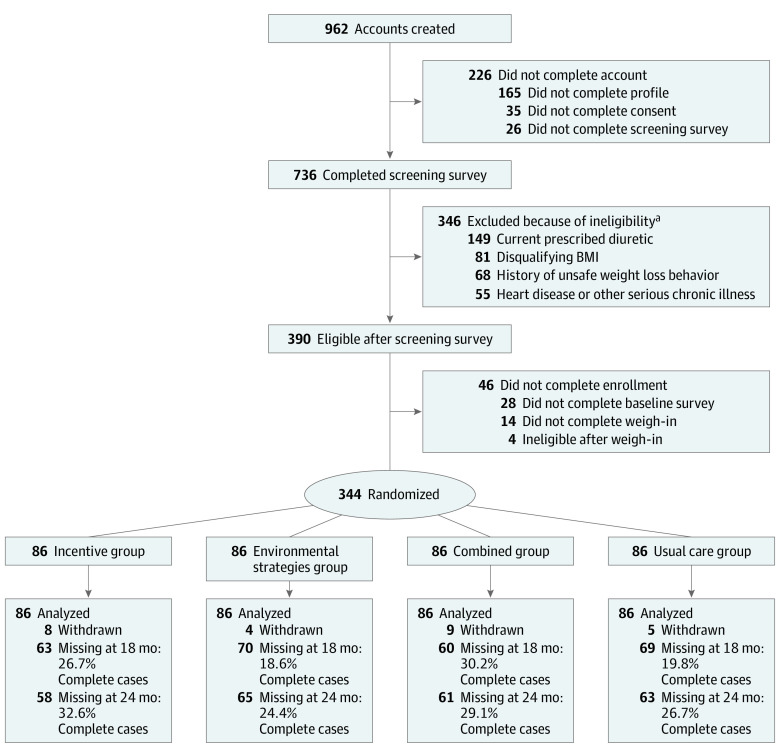
Healthy Weigh CONSORT Flow Diagram BMI indicates body mass index. ^a^These are the top 4 reasons for exclusion. Participants may have multiple reasons for ineligibility.

A total of 736 participants completed screening, 390 were eligible, and 344 were randomized across the 4 study groups, 86 in each group. Figure 1 shows the study flow diagram. Further details of recruitment were reported previously.^22^ Participant demographic characteristics are summarized in Table 1. Participants had a mean (SD) age of 45.6 (10.5) years and a mean (SD) BMI of 36.5 (7.1)^22^; 247 participants (71.8%) were women, 172 (50.0%) were Black, 14 (4.1%) were Hispanic, 138 (40.1%) were White, and 20 (5.8%) were other races and ethnicities, including Asian, Pacific Islander, American Indian, and multiracial. At the 18-month end point, 262 participants (76.2%) completed the study; dropout did not differ across study groups.

**Table 1.** Baseline Demographic and Other Characteristics for Participants

Characteristics	Participants, No. (%)				
	Total (N = 344)	Incentive (n = 86)	Environmental strategies (n = 86)	Combined (n = 86)	Usual care (n = 86)
Demographic characteristics					
Age, mean (SD), y	45.6 (10.5)	46.4 (9.5)	46.6 (10.9)	44.9 (11.1)	44.6 (10.6)
Sex					
Female	247 (71.8)	61 (70.9)	61 (70.9)	61 (70.9)	64 (74.4)
Male	97 (28.2)	25 (29.1)	25 (29.1)	25 (29.1)	22 (25.6)
Race and ethnicity					
Hispanic	14 (4.1)	4 (4.7)	2 (2.3)	4 (4.7)	4 (4.7)
Non-Hispanic					
Black	172 (50.0)	37 (43.0)	43 (50.0)	49 (57.0)	43 (50.0)
White	138 (40.1)	38 (44.2)	35 (40.7)	30 (34.9)	35 (40.7)
Other^a^	20 (5.8)	7 (8.1)	6 (7.0)	3 (3.5)	4 (4.7)
Education					
Less than college	19 (5.5)	5 (5.8)	4 (4.7)	5 (5.8)	5 (5.8)
Some college or special training	128 (37.2)	30 (34.9)	36 (41.9)	28 (32.6)	34 (39.5)
College graduate	197 (57.3)	51 (59.3)	46 (53.5)	53 (61.6)	47 (54.7)
Annual household income, $					
≤49 999	67 (21.4)	13 (17.1)	15 (19.2)	23 (29.5)	16 (19.8)
50 000-74 999	115 (36.7)	29 (38.2)	28 (35.9)	27 (34.6)	31 (38.3)
≥75 000	131 (41.9)	34 (44.7)	35 (44.9)	28 (35.9)	34 (42.0)
Household size, No. of individuals					
1-2	162 (47.1)	38 (44.2)	43 (50.0)	47 (54.7)	34 (39.5)
≥3	182 (52.9)	48 (55.8)	43 (50.0)	39 (45.3)	52 (60.5)
Personal characteristics and lifestyle					
Baseline body mass index, median (IQR)^b^	36.5 (7.1)	36.9 (6.1)	36.1 (7.3)	36.7 (8.7)	36.45 (7)
Stage of change, action and maintenance^c^	120 (34.9)	38 (44.2)	22 (25.6)	26 (30.2)	34 (39.5)
Moderate and vigorous physical activity, median (IQR), min/wk^d^^,^^e^	270 (540)	330 (480)	270 (620)	240 (600)	240 (435)
Walking, min/wk^d^^,^^e^	225 (375)	250 (330)	185 (310)	220 (430)	277.5 (510)
Cognitive restraint scale score, mean (SD)^f^	44.2 (16.8)	43.7 (17.7)	43.7 (17.5)	44.5 (17.1)	45.0 (15.2)
Eating scale score, mean (SD)^f^					
Uncontrolled	39.8 (18.6)	40.8 (19.1)	38.3 (18.6)	40.6 (18.3)	39.7 (18.7)
Emotional	46.5 (27.9)	48.1 (28.9)	46.6 (27.3)	46.8 (29.8)	44.6 (26.0)
SF-36 General Health score, mean (SD)^g^	64.4 (18.8)	67.4 (17.9)	62.7 (17.5)	62.0 (20.5)	65.7 (19.2)

Abbreviations: IQR, interquartile range; SF-36, 36-Item Short-Form Survey.

^a^ Includes Asian, Pacific Islander, American Indian, and multiracial individuals.

^b^ Body mass index is calculated as weight in kilograms divided by height in meters squared.

^c^ Stage of change has 2 categories: contemplation and preparation, and action and maintenance.

^d^ Measured using the International Physical Activity Questionnaire.

^e^ Data are missing for 31 participants for annual household income, 35 participants for moderate and vigorous physical activity, and 30 participants for walking.

^f^ Eating behavior control is measured by the Three Factor Eating Questionnaire. The raw eating scale scores are transformed to a 0 to 100 scale as follows: [(raw score – lowest possible raw score) / possible raw score range] × 100. Higher scores in the respective scales are indicative of greater cognitive restraint, uncontrolled eating, or emotional eating.

^g^ General health is assessed using SF-36 with default range from 1 to 100. Higher values denote better health conditions.

**Figure 2.**
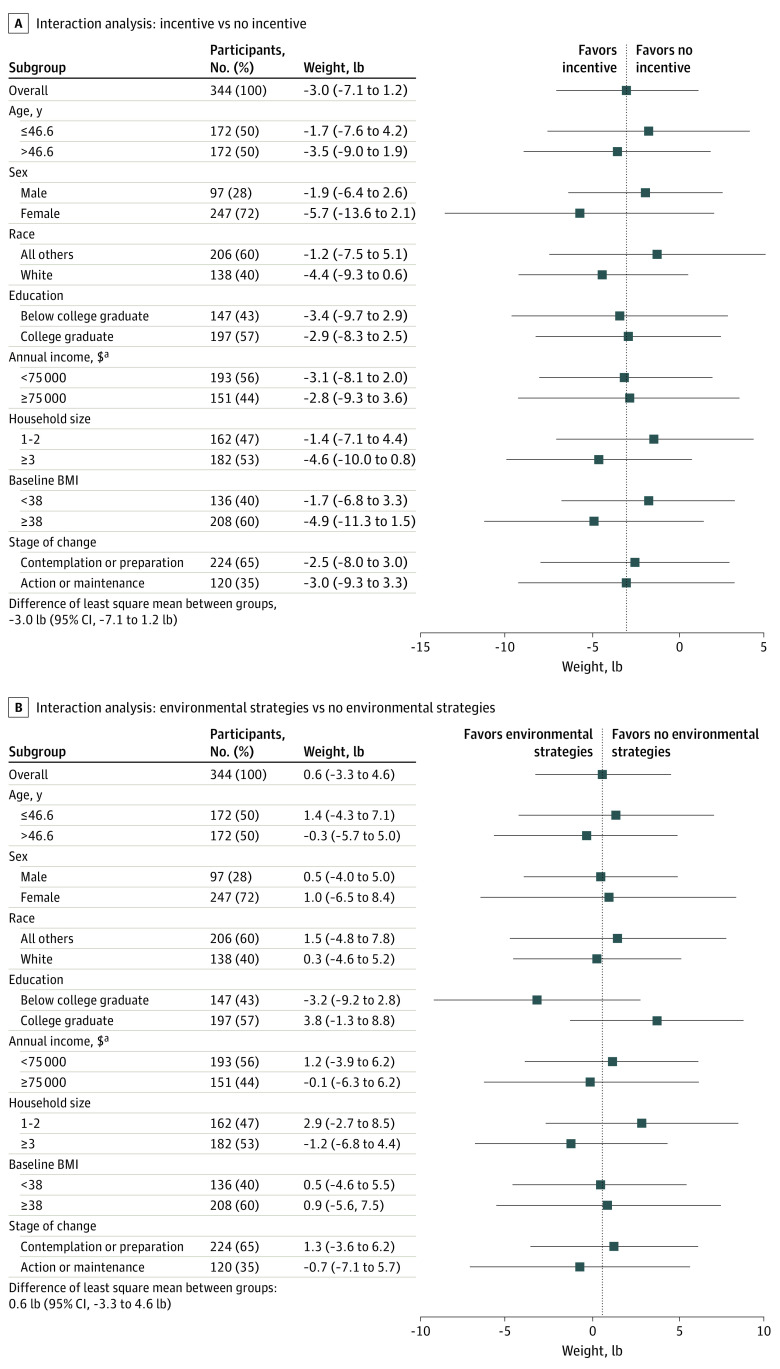
Interaction Analyses for Incentive Groups vs Nonincentive Groups and for Environmental Strategies Groups vs Non–Environmental Strategies Groups The interaction regression models are adjusted by the randomization strata variables of sex, employer, initial body mass index (BMI; calculated as weight in kilograms divided by height in meters squared); study group; and baseline participant characteristics of age, race/ethnicity, annual household income, education, baseline weight, marital status, household size, and stage of change. The main outcome shown in panel A is the difference of least square mean between incentive groups (incentive group plus combined group) and nonincentive groups (environmental strategies group plus usual care group). The main outcome shown in panel B is the difference of least square mean between environmental strategies groups (environmental strategies group plus combined group) and non–environmental strategies groups (incentive group plus usual care group). ^a^For annual household income, data are missing for 31 participants. For the intention-to-treat analysis, we preformed multiple imputation to the missing cases. The number of participants presented here is from the first iteration of multiple imputation.

At the primary end point of 18 months, none of the 5 primary pairwise group comparisons of interest for weight change from baseline was significant in the unadjusted analysis (Table 2). Comparing participants who received an incentive vs not, the unadjusted mean change from baseline was a loss of 2.8 lb (95% CI, −7.1 to 1.5 lb [mean, 1.26 kg; 95% CI, −3.20 to 0.68 kg]) more than control participants. For participants in an environmental strategies group vs not, there was a difference between groups in the change from baseline of 0.4 lb (95% CI, −3.7 to 4.4 lb [mean, 0.18 kg; 95% CI, −1.67 to 1.98 kg]). Adjusted results were similar with none of the primary pairwise comparisons significant at the Holm-adjusted level for multiple comparisons; however, the difference between incentive vs control was 5.4 lb (95% CI, −11.3 to 0.5 lb [2.45 kg; 95% CI, −5.09 to 0.23 kg]; unadjusted *P* = .07). Participants in the environmental strategies group lost a mean of a 2.2 lb (95% CI, −7.7 to 3.3 lb [mean, 1.00 kg; 95% CI, −3.47 to 1.49 kg]), and the combination group lost a mean of 2.4 lb (95% CI, −8.2 to 3.3 lb [mean, 1.09 kg; 95% CI, −3.69 to 1.49 kg]) more than participants in the usual care group. At 24 months, after 6 months without an intervention, the difference in the change from baseline was similar to the 18-month results, with no significant differences between groups for the adjusted or unadjusted pairwise or combined-group comparisons. All 3 intervention groups sustained approximately a 4 lb weight loss at 24 months. Similar results held true for the complete case cohort (eTable 1 in Supplement 2). There were no significant interactions for the incentive vs nonincentive groups by sex, age, race, education, income, household size, BMI, or stages of change (Figure 2A) or environmental strategy vs no–environmental strategy groups (Figure 2B).

**Table 2.** Analysis of the Main Effect for Weight Change at 18 and 24 Months Among Intention-to-Treat Population

Comparisons	Unadjusted analysis		Adjusted analysis^a^	
	Effect size, mean (95% CI), lb	*P* value	Effect size, mean (95% CI), lb	*P* value
From baseline to 18 mo				
Incentive vs usual care	−5.4 (−11.3 to 0.5)	.07	−5.5 (−11 to 0.0)	.05
Environmental strategies vs usual care	−2.2 (−7.7 to 3.3)	.43	−1.9 (−7.3 to 3.5)	.50
Combined vs usual care	−2.4 (−8.2 to 3.3)	.40	−2.3 (−8.1 to 3.6)	.44
Incentive vs combined	−2.9 (−9.1 to 3.2)	.35	−3.2 (−9.1 to 2.7)	.28
Environmental strategies vs combined	0.2 (−6 to 6.5)	.94	0.4 (−5.6 to 6.5)	.89
Incentive plus combined vs environmental strategies plus usual care	−2.8 (−7.1 to 1.5)	.20	−3.0 (−7.1 to 1.2)	.16
Environmental strategies plus combined vs incentive plus usual care	0.4 (−3.7 to 4.4)	.86	0.6 (−3.3 to 4.6)	.75
From baseline to 24 mo				
Incentive vs usual care	−4.7 (−11.1 to 1.7)	.15	−5.0 (−10.8 to 0.8)	.009
Environmental strategies vs usual care	−4.6 (−10.6 to 1.4)	.13	−4.4 (−10.2 to 1.3)	.13
Combined vs usual care	−4.8 (−11.2 to 1.6)	.14	−5.3 (−11.8 to 1.3)	.11
Incentive vs combined	0.1 (−6.6 to 6.8)	.98	0.3 (−5.9 to 6.5)	.93
Environmental strategies vs combined	0.2 (−6.1 to 6.5)	.95	0.8 (−5.3 to 7.0)	.79
Incentive plus combined vs environmental strategies plus usual care	−2.5 (−7.1 to 2.2)	.30	−2.9 (−7.4 to 1.6)	.20
Environmental strategies plus combined vs incentive plus usual care	−2.4 (−7.0 to 2.3)	.32	−2.4 (−6.9 to 2.1)	.30
From 18 to 24 mo				
Incentive vs usual care	0.7 (−6.4 to 7.7)	.85	0.5 (−6.2 to 7.2)	.88
Environmental strategies vs usual care	−2.4 (−8.5 to 3.6)	.43	−2.6 (−8.7 to 3.6)	.41
Combined vs usual care	−2.4 (−10.1 to 5.3)	.54	−3.0 (−10.7 to 4.7)	.44
Incentive vs combined	3.0 (−4.3 to 10.4)	.41	3.5 (−3.5 to 10.4)	.32
Environmental strategies vs combined	0 (−7.1 to 7.0)	.99	0.4 (−6.6 to 7.4)	.91
Incentive plus combined vs environmental strategies plus usual care	0.4 (−5.0 to 5.7)	.89	0.1 (−5.2 to 5.4)	.98
Environmental strategies plus combined vs incentive plus usual care	−2.7 (−7.6 to 2.2)	.27	−3.0 (−7.9 to 1.8)	.22

^a^ Generalized linear models are adjusted by the randomization strata variables of sex to employer and initial body mass index to study group to and baseline participant characteristics of age to race to annual household income to education to baseline weight to marital status to household size and stage of change.

eTable 2 in Supplement 2 shows the ITT intervention effects on the exploratory outcomes of total physical activity, cognitive restraint, uncontrolled eating, emotional eating, and SF-36 General Health score. For the 5 prespecified pairwise group comparisons, only SF-36 General Health score had a positive effect, with scores increasing by a mean of 5.6 points (95% CI, 0.7-10.6 points) in the environmental strategy group compared with the usual care group and 5.5 points (95% CI, 1.8-9.3 points) in the incentive group plus usual care group combined.

As shown in eTable 3 in Supplement 2, the per-protocol analysis found that the effect for the incentives-only group was larger than in the ITT analysis, and the effect for the environmental strategies–only group was also larger than in the ITT analysis, but the difference was not significant in adjusted analysis. Other comparisons (eFigure 1 and eFigure 2 in Supplement 2) were not significantly different between groups or combinations of groups in the adjusted analyses.

### Process Findings

**Figure 3.**
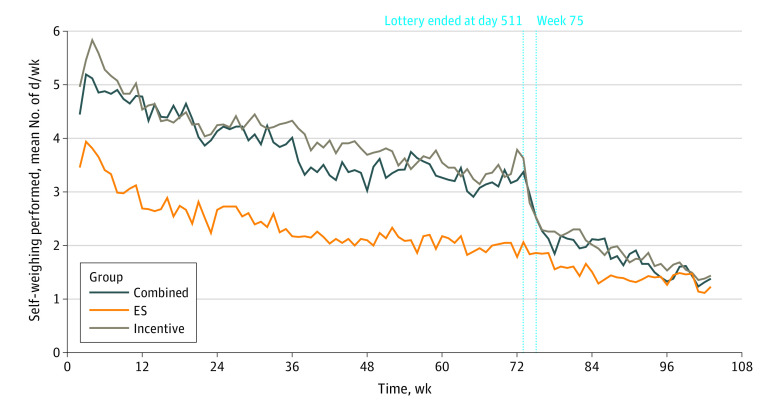
Days per Week Self-weighing Was Performed Among Study Participants Who Successfully Set Up the Scales Participants who did not have scales are excluded in the analysis, and those who withdrew are excluded from the denominator from the week they withdrew. Participants in usual care group are not included here because they did not have scales. The first week data are not included because it was a grace period for setting up the scale. The line of week 75 shows the wash-out period: 244 participants successfully set up their scales, including 79 in the incentive group, 82 in the environmental strategies (ES) group, and 83 in the combined group. At week 73, denominators were 73, 80, and 78 for the incentive, ES, and combined groups, respectively.

After the first week, there was a steady decrease in home self-weighing in all 3 groups with wireless scales (Figure 3); however, the 2 incentive groups weighed in more frequently than the environmental strategy group during the intervention period. In the generalized estimating equation model testing a time-treatment interaction, no significant differences were found between study groups in the rate of change over time (slope) of weigh-in frequency; however, participants in the incentive alone group and the combined group self-weighed a mean of 1.6 days per week (95% CI, 1.0-2.3 days per week) and 1.4 days per week (95% CI, 0.8-2.0 days per week), respectively, more often than the environmental strategies group. All 3 intervention groups had similar weekly weigh-in frequencies by 24 months (eTable 4 in Supplement 2).

The individually tailored text messages in the environmental strategies were well received. When asked whether they had used the tips to make recommended environmental modifications, a mean of 72% of respondents (range, 27%-96% of respondents) said they had tried each tip. Only 49 participants (28% of the environmental strategies groups) participated in the Facebook group.

Responses to the end-of-study survey (207 participants [60%]) indicated that most participants were satisfied with the study procedures (80% were very much or somewhat satisfied), and those who received wireless scales planned to use them after the study (82% said very much). Only one-third said they met their weight loss goal partly or completely. Some respondents indicated that they felt a need for more guidance or a more detailed weight loss plan.

## Discussion

The Healthy Weigh Study recruited a racially diverse, mostly female, employee population with obesity to a weight loss trial with 18 months of active intervention and a 6-month follow-up period. Participants were randomized to receive financial incentives for achieving weight loss goals, customized environmental strategies, a combined incentives and environmental strategies group, or usual care. At the end of active study period, none of the study groups achieved significantly greater weight loss than the usual care group, and the results were similar 6 months later. The highest weight loss occurred in the incentive groups, with 7.1 lb lost over 18 months. All 3 intervention groups sustained approximately a 4 lb weight loss at 24 months.

This study differs from previous employer-based weight loss trials rooted in behavioral economics, in that all participants had BMIs in the obese range (mean BMI, 36.5), and the intervention period was substantially longer than in previous studies. A systematic review of 47 workplace weight loss programs, most of them low-intensity as in this study, showed that cluster randomized trials of similar duration also found modest weight losses.^21^ Some short-term incentive interventions achieved greater initial weight loss of 10 to 14 lb^12,14^ that were not sustained after 4- to 8-month interventions.^34^ Other behavioral economics–based strategies, such as deposit contracts, achieved low participation rates.^35^ A recent systematic review of strategies to improve workplace policies and environments to reduce risk behaviors, including obesity, noted that the available evidence is “sparse and inconsistent.”^36^

### Strengths and Limitations

This study had both strengths and limitations. The use of wireless scales to objectively track weight over time is a strength, and as is the diverse study sample. Limitations include the study being conducted in urban workplaces without cafeterias and possible selection bias by study participants. It is possible that the design of the incentives made them less effective than we had hoped.^37^ There was a delay between earning incentive payments and receiving the funds. Also, general study payments for enrollment and completion of measures totaled $300 across the course of the study, potentially muting the incremental effect of offering incentives tied to weight loss.

A recurrent theme in end-of-study surveys was that participants felt they would have benefited from more intensive guidance, such as ongoing counseling or coaching. Also, environmental manipulations may need to be more intensive to promote greater weight loss success. Furthermore, the power calculations for this study assumed a higher average weight loss than was achieved in the intervention groups.

## Conclusions

Both initial and sustained weight loss remain substantial challenges to improve health and reduce risk of major chronic diseases. In the Healthy Weigh trial, incentives and environmental strategies led to modest but nonsignificant improvements in weight loss. From a translational standpoint, benefits designs could consider incorporating ongoing financial incentives for weight loss among employees with obesity, while linking online support to more intensive personalized interactive approaches.

## Notes

Supplement 1.
Trial Protocol

Supplement 2. **eTable 1.** Analysis of the Main Effect for Weight Change (lbs.) at 18 and 24 Months in the Complete Case Population **eTable 2. **Unadjusted Analysis of Exploratory Outcomes and Potential Mechanisms of Change Between Baseline and 18 Months Among Complete Case and Intention-to-Treat Populations **eTable 3.** Analysis of the Main Effect for Weight Change (lbs.) at 18 Months in Per-Protocol Analysis Population **eTable 4.** Analysis of Frequency of In-Home Weigh-In per Week by Intervention Phase (N=244) **eFigure 1.** Mean of Weight Change (lbs.) From Baseline to Different Time Points by Arm in Intention-to-Treat Population (N=344) **eFigure 2. **Mean of Weight Change (lbs.) From Baseline at Different Time Points by Arm in Complete Case Population

Supplement 3.
Data Sharing Statement
