# Supplementary material for: Effect of Financial Incentives and Environmental Strategies on Weight Loss in the Healthy Weigh Study: A Randomized Clinical Trial
Source: JAMA Netw Open. 2021 Sep 7;4(9):e2124132. doi: 10.1001/jamanetworkopen.2021.24132 (PMC8424479; doi:10.1001/jamanetworkopen.2021.24132)
Supplement: Supplement 1. — Trial Protocol [file jamanetwopen-e2124132-s001.pdf]

A Randomized Controlled Trial of Incentives vs Environmental Strategies for Weight Loss

Study Protocol

v August 3, 2015

1. Abstract 4
2. Background 4
   2.1. Impact of Obesity on Health, Benefits of Weight Loss
   2.2. Changes in benefit design and the use of incentives for employees
   2.3. Use of Financial Incentives to Change Health Behaior in General
   2.4. Effects of Financial Incentive Interventions for Obese Patients
   2.5. Impact of Environmental Changes on Food intake and Physical activity
   2.6. Cost of Obestiy and Cost-effectiveness of Behavioral Interventions
3. Data Management/Way to Health Platform 7
4. Overall Objectives/ Specific Aims 8
   Primary Aims
      1. Lottery vs. Control Group
      2. Environmental Strategy vs. Control Group
      3. Combined Incentive and Environmental Strategy vs. Control Group
      4. Combined Incentive and Environmental Strategy and Weight loss over 18 months
   Secondary Aims
      5. Assess costs by arm
5. Primary Outcome Variable 9
   5.1. Change in weight from BL to 18M
   5.2 Specific Aims 1-3
   5.3 Specific Aim 4
6. Secondary Outcome Variable(s) 9
   6.1 Specific Aim 5

7. Study Instruments 9
7.1 Surveys and Assessments
7.2 Demographic Measures
7.3 Physical Activity
7.4 Dietary Behavior
7.5 Stages of Change Model
7.7 Biometric Assessment Data Part A
7.8 Biometrics Assessment Data Part B
7.9 Medical History
7.10 Health Related Quality of life
7.11 Healthy Utility Index (HUI)
7.12 Insurance Claims
7.13 Withings Scale
8. Study Design 10
8.1. Research Design and Methods
8.2. Measurement of cost effectiveness
8.3. Key Inclusion Criteria
8.4. Key Exclusion Criteria
9. Subject Recruitment 13
9.1. Target Population
9.2. Accrual
9.3 Subject Recruitment
9.4 Subject Compensation
10. Study Procedures 14
Consent Process
10.1 Overview
Procedures
10.2 Baseline pre-treatment assessment for patients
10.3 Randomization

10.4 Environmental Change Strategies
10.5 Design of the Incentives
10.6 Study Duration
10.7 Dissemination
11. Analysis Plan 18
11.1 Statistical Considerations
11.2 Power and Sample Sizze Consideration
11.3 Measurement of Effect
12. Human Research Protection 21
12.1 Subject Confidentiality
12.2 Subject Privacy
12. 3 Protection of Human Participants
12.4 Cost Effectiveness Analysis
12.5 Source of Materials
12.6 Potential Risks Involved in the Proposed Study
12.7 Risks Involved in the Cost-Effectiveness Analysis
12.8 Adequacy of Protection from Risks Recruitment and Informed Consent
12.9 Protection against risk
12.10 Electronic Data Security
12.11 Potential Study Benefits
12.12 Resources necessary for Human Research Protection
13. Data Protection 27
13.1 Data and Safety Monitoring Plan
13.2 Data and Safety Monitoring Board
13.3 Potential Study Benefits
13.4 Alternatives to Participation
13.5 Risk/Benefit Assessment

1. Abstract

Identifying effective strategies for treating obesity is both a clinical challenge and a public health priority. Between 1960 and 2010, obesity prevalence (body mass index [BMI] 30 kg/m2) in US adults increased from 11% to 35.5% in men and from 16% to 35.8% in women, and about 70% of US adults are considered either overweight or obese. This situation has troubling implications, as obesity in young adulthood and middle age translates into higher rates of cardiovascular risk factors, disability, hospitalization, Medicare expenditures, and mortality risk later in life. While a variety of approaches are successful in achieving initial weight loss techniques for combining initial weight loss and successful maintenance of weight loss have been elusive. Research shows that most people who successfully lose weight will regain 1/3 to 2/3 of that weight after one year. Rigorous testing of promising, innovative approaches to weight loss and maintenance is an important public health priority. While both behavioral economic and environmental strategies have shown promise, we propose to test a novel blend of these approaches in this initiative. We will also test these interventions in populations with a high rate of African Americans and low to moderate income individuals who are obese, addressing important concerns about the health disparities due to high rates of obesity. The proposed study will evaluate the comparative effectiveness of behavioral economic financial incentives and environmental strategies, separately and together, in achieving initial weight loss and maintenance of weight loss, in obese employee populations. Our study contributes to the CDCs efforts to combat obesity but in particular to CDCs winnable battles (physical activity and obesity); the NCCDPHP strategic priorities around well-being, health equity, and evaluation and dissemination of environmental and systems-wide solutions to address public health problems; and the NCCDPHP domain around healthier worksite initiatives, CDC efforts to improve nutrition and physical activity to prevent obesity, and reduction of cardiovascular disease risk. We propose a 4-arm randomized controlled trial (RCT) in which 328 employees of the City of Philadelphia, IBC, and SEPTA with initial Body Mass Index of 30 or greater and at least 1 other cardiovascular risk factor (high blood pressure, hypercholesterolemia, diabetes, smoking) will be randomized to receive one of the following: 1) Daily lottery type incentives tied to achievement of weight loss goals (incentive arm); 2) individually tailored environmental strategies around food intake and physical activity (environmental arm); 3) a combination of incentives and environmental strategies (combined arm); or 4) standard employee wellness benefits and weigh-ins every 6 months (control arm). Phase I of the study (first 6 months) will focus on weight loss; Phase II (months 7-18) will focus on continued weight loss or maintenance for those who choose to maintain weight loss as opposed to continuing to try to lose weight; Phase III (months 19-24) will provide a period of post-intervention follow-up to measure sustainability of effects.

2. Background

2.1 <u>Impact of Obesity on Health, Benefits of Weight Loss.</u> Obesity is the second leading cause of preventable death in the United States, associated with high blood pressure (BP), type 2 diabetes, coronary heart disease, and osteoarthritis. Weight loss of just 5-10 kg can improve risk factors (e.g., BP, glycemia, and serum lipid levels) and reduce the incidence of diabetes. In older adults, weight loss interventions have shown clear improvements in BP, arthritis, and functional status. However, maintenance of weight loss is needed to achieve long term health benefits, and successful strategies for

long-term maintenance are lacking. Weight regain after a period of intentional weight loss is widely observed with all interventions due to loss of motivation, lack of sustained rewards for weight loss behavior, difficulty adhering to diet, and need for ego-depleting exertion of willpower. Since weight loss programs offered as part of standard employer-sponsored benefits are generally unsuccessful in achieving weight loss maintenance, supplemental motivation from incentives or environmental feedback may help people keep weight off.

2.2 Changes in benefit design and the use of incentives for employees. There have been significant shifts in recent years in the use of incentives to increase healthy behaviors among employees of American companies. HIPAA's 2006 regulations permitted employer health plans to offer financial incentives for achieving health- contingent standards such as body mass index as long as this was part of a wellness program. Programs were required to meet a number of conditions, including that they have a "reasonable chance" of improving health and not be "overly burdensome. The Affordable Care Act (ACA) increased the incentive ceiling to 30% of coverage costs and gave regulators the authority to further increase the ceiling to 50% if "appropriate." In recently issued final rules, regulators lifted the ceiling to 50% as long as any incentives beyond the 30% threshold targeted tobacco use.99 In 2013, approximately 85% of large employers in the US are using incentives for healthy behavior, making the use of such approaches increasingly a widely used standard for efforts to reduce obesity. However, the approaches being utilized generally have not been well tested and rarely utilize insights from behavioral economics that utilize the decision errors that are common in people to design more effective programs. These strategies also fail to incorporate environmental change strategies which are increasingly recognized as important to obesity reduction and maintenance of healthy weight, and few such strategies have been rigorously evaluated in the context of weight management programs.

2.3 Use of Financial Incentives to Change Health Behaviors in General. Individuals put disproportionate value on present relative to future costs and benefits, known as present-biased preferences. While this bias typically works against healthy behavior, the same factors can be used to promote healthy behavior by providing tangible but small immediate rewards for beneficial behaviors. Evidence from hard-to-change behaviors such as tobacco and other substance use, indicate that incentives can be beneficial, but patients require regular monitoring, and frequent feedback is an important component to success. A review of 11 randomized trials of financial incentives found that financial incentives promoted adherence better than any tested alternative, leading to better blood pressure control, appointment attendance, and higher immunization rates. Other reviews have found various incentives (including lotteries) effective in changing behavior. However, few studies have examined longer-term effects of incentives on health behaviors after cessation of incentives, or the relative effectiveness of incentives vs. other obesity reduction strategies. Lottery-type incentives are particularly appealing for improving adherence to a weight loss regimen. Although small payouts (e.g.,$10 to quit smoking) may not be effective, lottery incentives have shown promise in altering health behaviors even when awards have relatively low expected values (e.g., $50). Lotteries typically include several features that make them so appealing, despite a very low rate of return. They typically offer a combination of a relatively large chance of a small payout and a very small chance of a larger payout. The frequent small payoffs increase the attractiveness of lotteries by giving lottery players intermittent positive reinforcement. Moreover,

the feedback is often very rapid; most games have daily draws and more than 40% of state revenues now come from instant scratch-off tickets. The small chance of a large payoff is especially attractive because people tend to overweigh small probabilities in making decisions. Structuring financial incentives as a lottery has several benefits over a set pay-off amount. Lotteries may be less costly to provide than comparable cash incentives because of the effectiveness of intermittent reinforcement. Beyond their monetary payoffs, lotteries provide entertainment value, enhancing their reinforcing properties as a motivator. Lotteries may be particularly effective in lower-income populations (in whom lotteries are more popular), who have higher rates of non-adherence than other populations.

2.4 Effects of Financial Incentive Interventions for Obese Patients. Incentives are effective in weight loss interventions. Jeffery et al showed that participants without any weight management training lost weight when they deposited valuables with a therapist with return of their valuables contingent on progress towards pre-specified goals, and that incentives for weight loss are more effective than incentives for attending weight loss training. Finkelstein et al. found that over a 3- month period, participants offered $14 per percentage point of weight loss lost 4.7 pounds whereas participants offered $7 lost 3.0 pounds and control participants lost 2.0 pounds. In work done by our group using daily lotteries (published in JAMA), we found that about 50% of participants offered an incentive reached a weight loss goal of 1 pound per week over 16 weeks (average weight loss 13.1 pounds) compared to about 10% of control group participants (average weight loss 4.0 pounds). Dropout rates were about 7%, remarkably low for a weight loss intervention. Further work extended successful weight loss to 8 months and showed the effectiveness of competition between groups in augmenting motivation, though the individual arm was less effective without frequent feedback. These studies illustrate that incentives ideally provide frequent feedback, and that daily lottery incentives using a technology platform can be highly effective at sustaining engagement and achievement of weight loss goals.

2.5 Impact of Environmental Changes on Food intake and Physical activity. It has long been recognized that social and physical environmental factors influence food intake and physical activity. Worksite interventions to improve food environments have shown modest success. Most worksite nutrition and weight intervention studies have not included clearly defined environmental strategies, though combination interventions have achieved significant, albeit modest, success. The use of environmental interventions to increase physical activity is recommended, though the evidence base of intervention research is limited. Cross-sectional research indicates that having multiple environmental supports (stairways, facilities, equipment) at worksites is associated with meeting physical activity interventions. In addition to environmental changes to worksite structures, environmental re-engineering is a promising strategy and is being studied in several trials with a home environment focus. A recent study of environmental changes for weight gain prevention in worksites found no impact on body mass from environmental changes alone, perhaps due to limited strength of the interventions, deployment of single strategies in isolation, inadequate implementation, and because such studies included normal weight, overweight, and obese employees.

2.6 Cost of Obesity and Cost-effectiveness of Behavioral Interventions. Recent studies concluded that annual US expenditures attributable to overweight and obesity are between $90 and $100 billion.

Among nationally representative cohorts, the average annual incremental cost of obesity is about $1,308. Higher BMIs in young adulthood and middle age are associated with substantially higher Medicare expenditures in old age. Higher BMI also translates into significantly higher health care charges within 18 months, 1 suggesting that health plans may find it cost effective to invest in behavior modification to lower health expenditures. Several economic assessments of behavioral interventions for weight loss have been published. The Diabetes Prevention Program, reported that, compared to placebo, the intensive lifestyle intervention had cost per QALY ratios ranging between $32,000 and $52,000, depending upon whether direct medical costs alone or direct and indirect costs were counted. Finkelstein et al. reported that the 1-year incremental cost per 1% reduction in 10-year CHD risk associated with an enhanced behavioral intervention was $637.135 The substantial health and economic consequences of obesity highlight the importance of testing new approaches to maintenance of weight loss and the potential cost effectiveness of successful interventions.

3. Data Management and the Way to Health Platform

The Way to Health online platform will serve as the core mechanism for recruiting and enrolling subjects, transmitting general and intervention-specific messages, collecting individual weight measurements and online survey data, and providing regular feedback to subjects on their progress in the study. This platform has supported a series of other NIH-funded studies that harness new technologies to encourage healthy behaviors and treatment adherence. The University of Pennsylvania Biomedical Informatics Consortium (BMIC) will be the hub for the hardware and database infrastructure that will support the project and where the web portal is based. The data collected for this study will be stored in mySQL databases on a dedicated BMIC-run server. All data will be stored in both a non-editable database and in a separate modifiable database, allowing researchers to correct mistakes while preserving the raw data for auditing purposes. Every SQL transaction, including accessing and changing data, will be logged for auditing purposes. Data will be entered into the database through several different mechanisms. Participants will enter their own personal information and respond to surveys through a PHP-based web interface. Researchers will have a separate interface that will allow them to manually enter data if needed. The dataset will be blinded of all personally identifiable information when exported for analysis. The web application will automatically remove all identifiers when a researcher requests an analytic dataset. The only people with access to identifiable participant information will be pre-specified research staff responsible for contacting participants for follow-up. Personal information and research data will be stored in separate SQL tables and will be linked by a computer-generated ID number. Furthermore, the web platform will be set up with pre-specified ranges of eligible values for most questions to minimize participants' data entry errors. Specifically designated administrators will have the ability to make corrections. Each modification, however, will be logged along with justification for the change. The original data will be preserved in a separate non-modifiable database. The UPENN Biomedical Informatics Consortium (BMIC) will be the hub for the hardware and database infrastructure that will support the project and where the Way to Health web portal is based. The BMIC is a joint effort of the University of Pennsylvania's Abramson Cancer Center, the Cardiovascular Institute, the Department of Pathology, and the Leonard Davis Institute. The BMIC provides a secure computing environment for a large volume of highly sensitive

data, including clinical, genetic, socioeconomic, and financial information. Among the IT projects currently managed by BMIC are: (1) the capture and organization of complex, longitudinal clinical data via web and clinical applications portals from cancer patients enrolled in clinical trials; (2) the integration of genetic array databases and clinical data obtained from patients with cardiovascular disease; (3) computational biology and cytometry database management and analyses; (4) economic and health policy research using Medicare claims from over 40 million Medicare beneficiaries. BMIC requires all users of data or applications on BMIC servers to complete a BMIC-hosted cybersecurity awareness course annually, which stresses federal data security policies under data use agreements with the university. Curriculum includes HIPAA training and covers secure data transfer, passwords, computer security habits and knowledge of what constitutes misuse or inappropriate use of the server. We will implement multiple, redundant protective measures to guarantee the privacy and security of the participant data. All investigators and research staff with direct access to the identifiable data will be required to undergo annual responsible conduct of research, cybersecurity, and Health Insurance Portability and Accountability Act certification in accordance with University of Pennsylvania regulations. All data for this project will be stored on the secure/firewalled servers of the BMIC Data Center, in data files that will be protected by multiple password layers. These data servers are maintained in a guarded facility behind several locked doors, with very limited physical access rights. They are also cyber-protected by extensive firewalls and multiple layers of communication encryption. Electronic access rights are carefully controlled by University of Pennsylvania system managers. We will use highly secure methods of data encryption for all transactions involving participants' financial information using a level of security comparable to what is used in commercial financial transactions. We believe this multi-layer system of data security, identical to the system protecting the University of Pennsylvania Health Systems medical records, greatly minimizes the risk of loss of privacy.

4. Overall Objectives/Specific Aims

<u>Primary</u>

1. Assess the effectiveness of a daily lottery-based financial incentive, relative to the control group, on cumulative weight loss over an 18-month period. *H1: Mean weight loss from baseline to 18 months among participants randomized to the lottery incentive will be at least 5 kg greater than among control group participants.*

2. Assess the effectiveness of environmental strategies, relative to the control group, on cumulative weight loss over an 18-month period. *H2: Mean weight loss from baseline to 18 months among participants randomized to the environmental strategies group will be at least 5 kg greater than among control group participants.*

3. Assess the effectiveness of the combined incentives and environmental strategies arm, relative to the control group, on cumulative weight loss over an 18-month period. *H3: Mean weight loss from baseline to 18 months among participants randomized to the combined arm will be at least 8 kg greater than among control group participants.*

4. Assess the comparative effectiveness of a combination of lottery-based financial incentives and environmental strategies on cumulative weight loss over an 18-month period. *H4: Mean*

weight loss among participants randomized to the combined arm will be at least 3 kg greater than among participants in either of the individual arms.

Secondary

5. Assess costs of each of the intervention arms from both the employer and social perspective and compare cost differences between each arm relative to effectiveness measured by incremental weight loss achieved. *H5: From an employer's perspective, each individual intervention will have favorable cost-effectiveness ratios relative to control; the combined arm will have a favorable cost-effectiveness ratio relative to individual arms.*

5. Primary Outcome Variable

5.1 Change in weight from baseline to 18 months.

5.2 Specific Aims 1-3

Compare effectiveness of financial incentive, environmental strategy, and combined arm to control during Phases I and II. For expected weight change between months 0-18 of -8 kg, -5 kg and 0 kg in the combined, intervention and control groups, respectively (a net difference between single intervention and control groups of 5 kg and between combined intervention and control groups of 8 kg) and assuming a standard deviation (S.D.) of weight loss of 5 kg, 65 participants per group provide 90% power to detect at least one of these contrasts. The sample size provides 80% power to detect a difference of 4.6 kg in weight change between single intervention and control groups and 7.3 kg between the combined and control groups.

5.3 Specific Aim 4

Compare effectiveness of financial incentives and environmental strategies and combined arm during Phases I and II.

6. Secondary Outcome Variable(s)

Specific Aim 5

6.1 Compare the cost differences between each arm relative to the effectiveness measured by incremental weight loss achieved.

7. Study Instruments

7.1 Surveys/Assessments will be administered during various time points throughout the study via the online Way to Health (WTH) study platform

7.2 Medical history variables will be obtained as part of eligibility screening on the WTH study website.

7.3 Demographic measures will be collected at baseline (race, income, household size, education).

7.4 Physical Activity: The Global Physical Activity Questionnaire (GPAQ) measures adherence to national recommendations for moderate (30 minutes moderate physical activity 5 days per week) and vigorous (20 minutes continuous vigorous activity on 3 days per week) physical activity recommendations. This will be collected at 0, 6, 12, 18, and 24 months.

7.5 Dietary Behavior: The Food Intake 3 Factor Eating Questionnaire will be collected at months 0, 6, 12, and 18, and 24.

7.6 Stages of Change Model [Transtheoretical Model (TTM)], a commonly used measure of behavioral change which envisions the process of behavior change as stages through which an individual may progress, regress, or cycle as needed. This correlates with many types of health behaviors within different populations. This will be measured at 0, 6, 12, 18, and 24 months. 7.7 As an ongoing measure of health-related quality of life we will use the SF-8 form (shortened version of SF-36, to assess quality of life in different domains in the general population) to reduce data collection and respondent burden. The SF-8 form will be administered at 0, 6, 12, 18, and 24 months.

7.8 We will use the EuroQol survey at 0, 6, 12, 18, and 24 months. The EuroQol survey is an assessment for overall quality of life.

7.9 Biometric assessment data, medical benefit claims information, and wellness program activities data:

Biometric assessment data will be obtained as part of their routine biometric assessments by the participant's employers (or 3rd party wellness vendors who provide wellness services for employees of IBC, the City of Philadelphia, and SEPTA), all of whom share the same insurance carrier, Independence Blue Cross. {height; weight; blood pressure; cholesterol; glucose; HbA1c (if applicable to participant). The opportunity to have these data collected for free are available to employees at SEPTA, IBC, and the City of Philadelphia as part of annual wellness biometric screening. Approximately 30% of all employees participate in biometric screening. We will use this as a secondary measure for exploratory analyses.

Medical benefit claims information will be collected from the participants’ insurer, Independence Blue Cross, which is relevant to our research.

Information on the participants’ use of wellness program activities provided by the employers will be collected (the City of Philadelphia, IBC, SEPTA).

- The use of wellness program activities and biometric screening results will only be collected from the time participants enroll in the study until their completion date of the study. The medical benefit claims information will be collected starting from one year prior to participants’ enrollment in the study, until their completion date of the study.
- The three categories of data listed above (biometric assessment data, medical benefit claims data, and wellness program activities) will be included in the informed consent form. In

addition, all necessary Data Use Agreements, including the method of transfer to the University of Pennsylvania, will be executed and communicated to the IRB prior to the transfer of all data.

7.10 Withings Scale: Digital scales will be distributed to subjects in this study to measure and electronically deliver subjects' weight measurements throughout the study using a secure wireless connection.

8. Study Design

8.1 Research Design and Methods

Study participants will be obese employees recruited from three employers: SEPTA, IBC, and the City of Philadelphia. We will structure the first 6 months of the study (Phase I) as a weight loss phase in which participants will be encouraged as a weight loss goal to lose 0.5 pound per week [Note: weight loss targets for sample size calculations use kg to fit in with the literature but we will use pounds for the incentive targets, as that is what Americans are most familiar with]. Phase II (months 7-18) will consist of a period of weight loss maintenance in which participants will be able to choose a weight loss goal each month of either 0 or 0.5, pounds of weight loss per week. Phase III (months 19-24) will be a period of post-intervention follow-up concluding with an in-person weigh-in at month 24.

Participants will be randomized to either a control group (information provision and the standard wellness program at each employer along with measurement of weight at 0, 6, 12, 18, and 24 months), a lottery-based financial incentive, or a series of environmental strategies and counseling regarding food intake and physical activity, or a combination of the financial incentive and environmental strategies. Participants in the incentive arm will be eligible for daily winnings based on the weights that are transmitted using wireless scales which we will provide so that they receive daily feedback on their winnings to keep weight loss goals salient. However, we will only provide the full payments based on validation of weight measurement at 6 months intervals, taking advantage of the motivating power of loss aversion by highlighting to participants that they only receive their daily winnings if they continue to maintain weight loss (or lose further weight) (an approach we have used with great success in previous studies).

All participants will receive $50 after validation of weight measurements at months 0, 6, 12, 18 and 24, regardless of what their weight is, an approach we have used in many studies to minimize the risk of differential rates of lost to follow-up in the intervention and control arms. The study is powered to test the effectiveness of each intervention arm relative to control as well as the relative effectiveness of the intervention arms. A cost-effectiveness analysis will be conducted to examine potential for wider dissemination and implementation. Measures and time points are shown in Table 1 (attached with the protocol application). We will leverage existing annual biometric assessments done by each of our employer partners at baseline, 12 months, and 24 months for information on glucose, cholesterol, and blood pressure. Dependent measures for primary aims: We will use change in weight as the primary outcome measure. This will match our primary outcome with the tracking that participants are doing on their own at home using wireless scales.

Measures of mechanisms for degree of weight change: Approaches to assessing measures related to daily weight tracking, dietary behavior, physical activity, and psychosocial constructs are as follows: Ongoing tracking of participation: For all participants we will use the Way to Health system to track participation in their respective arm: (1) daily weigh-ins and receipt of incentives (incentive and combined arm); (2) daily weigh-ins and use of environmental strategies at home or at work (environmental, incentive, combined arm); (3) study follow-up visits attended regardless of arm. The Way to Health system will track this using a substantial degree of automation. In the Environmental and Combined arms, use of recommended strategies will be tracked by logs as described above. If necessary, a stepped approach to greater promotion of the strategies will be used to ensure adequate uptake or dose of the environmental strategies

8.2 Measurement of cost-effectiveness

We will conduct a within-trial analysis that directly compares incremental costs and maintenance of weight loss using data measured in the trial. The principal analyses will compare over 18 months the incremental cost effectiveness ratios estimated from the incremental costs and effects between each of the individual arms and the control group as well as the combined intervention versus each individual arm. The analysis will be from the employer perspective, as the employer faces the decision as to whether to implement this program. Secondary analyses will evaluate the same incremental cost-effectiveness ratios but: 1) use data following 6 months of post-intervention follow-up (24 month visit); 2) use data at 12 months, a common follow-up interval in employee health programs; and 3) use the limited social perspective (because participant costs are crucial to understanding the full economic impact of interventions). Measurement of cost: For the employer perspective, measured costs will include: 1) incentive payments; 2) administrative costs of providing the interventions; and 3) medical expenditures. For the limited social perspective, measured costs will include 1) the costs included in the employer's perspective, 2) exercise costs, and 3) costs of nonstudy counseling services. This social perspective is limited in that we include incentive payments (which are transfers and typically omitted from the social perspective) and federal fee schedules to cost out medical services (as a proxy for social opportunity cost). Service use already documented in the study: Many of the services that will be costed out are already being measured in the clinical case report forms detailing participant adherence to daily feedback, lottery winnings, attendance and participation, dietary behavior, and physical activity. Project personnel diaries: Project personnel will complete time-diaries quarterly detailing their time spent on administrative tasks in the past week including time administering all participant related aspects of the intervention and usual care (excluding time related to general project administration). These tasks will be itemized by type of activity. They will also record the number of participant contacts by type of activity. Using personnel wages, these dairies will be used to estimate unit costs of administrative contacts. Elements of cost: 1) Incentive payments. Incentive payments to participants will be computed directly from the amount of incentive paid in the trial. The costs will include the daily lottery payments and the direct payments. 2) Administrative costs of program: The administrative costs of the incentive and environmental strategies interventions will include those for maintaining account balances, mailing payments and conducting follow-up visits, and other costs such as the scales used to weigh the participants. We will include project personnel costs by measuring the

average time required for project personnel to conduct specific intervention-associated administrative tasks as opposed to general project/ evaluation administration using personnel wages to convert this administrative time to costs.

8.3 Key Inclusion Criteria

Eligibility Criteria: Men and women ≥ 18yrs. of age who are full-time or part-time employees at SEPTA, IBC, or the City of Philadelphia; who have a BMI of 30 to 55 kg/m2. (the upper limit of BMI will minimize the influence of outliers on the main result of weight loss). Participants must also have at least one other cardiovascular risk factor (high blood pressure, hypercholesterolemia, diabetes, smoking), which will be obtained by self-report. Participants must receive their health benefits from Independence Blue Cross; one of our partners in the study.

8.4 Key Exclusion Criteria

Exclusion criteria will be limited to factors that may confound results or that make participation in a weight loss program unfeasible, unsafe, or require more intensive monitoring, as detailed in our prior work. These exclusions include: unstable heart disease, uncontrolled hypertension, kidney disease, and other serious chronic illness (e.g., transplant recipient, terminal illness); substance abuse; bulimia nervosa or related behaviors; or diabetes medication other than metformin; pregnancy or breast feeding; contraindications to counseling about diet, physical activity, or weight reduction; unstable mental illness. Participants with diabetes on medications besides metformin are excluded because these medications may require adjustment during weight loss to prevent hypoglycemia and dehydration. Individuals unable to read consent forms or fill out surveys in English will be excluded.

9. Subject Recruitment

9.1 Target Population

For this study we are collaborating with 3 important community partners in Philadelphia: SEPTA (which runs public transit in Philadelphia); IBC, the region's largest insurer; and the City of Philadelphia, the largest employer in Philadelphia. Each of these entities has a large number of employees (9,200 SEPTA; 4,500 IBC; and 5,300 City of Philadelphia) with a high degree of obesity (estimated 55% SEPTA, 36% IBC, 42% City); and a high proportion of African Americans (56.6% SEPTA, 38.4% IBC, 50% City). Eligible participants will be men and women above age 18 who are full-time or part-time employees at SEPTA, IBC, or the City of Philadelphia who have a BMI of ≥30 to ≤ 55 kg/m2. Participants must also have at least one other cardiovascular risk factor (high blood pressure, hypercholesterolemia, diabetes, smoking), which will be obtained by self report.

9.2 Accrual

There are an estimated 4,800 employees from the Southeastern Pennsylvania Transportation Authority (SEPTA), Independence Blue Cross (IBC), and the City of Philadelphia who have BMI greater than 30 and 1 or more cardiovascular risk factor, of whom approximately 48% are black. We plan to enroll 328 participants over 6 months making our recruitment targets easily attainable given the size of the recruitment pool.

9.3 Subject Recruitment

With assistance from IBC, SEPTA, and the City Department of Public health, we will advertise this as a research study of different ways to help people lose and keep off weight. The initial outreach to employees will be facilitated by IBC, SEPTA, and the City of Philadelphia, utilizing their existing communication channels (e.g. letter mailings, email, text, intranet, flyers, posters, digital display in the workplace). All recruitment materials will be submitted to the IRB before any study activities occur.

Interested participants will be asked to go to the Way to Health (WTH) website for prescreening and eligibility ascertainment. The screening intake form will be completed using the WTH website supplemented by phone support as needed. In order to verify BMI eligibility for the study, a verified in-person weight measurement will be required during the screening process.

Recruitment at Independence Blue Cross site (IBC):

After initial outreach by IBC's communication channels, individuals will be asked go to the Way to Health (WTH) website for prescreening and eligibility ascertainment. In collaboration with IBC, the research study team will have a table set up at the IBC site during their annual biometric screening event. For IBC employees who previously completed online pre-screening and consent, the research staff will obtain individuals' weight measurement from their biometric screening. For IBC employees who did not complete the online pre-screening and consent prior to the biometric screening event, verbal consent will be obtained (similar to phone screening) to record their weight measurement from the biometric screening in order to determine BMI eligibility for the study.

For IBC employees who are interested in the study but did not attend the biometric screening event, in-person weight measurements will be obtained on site by the Fitness Director at IBC's MediFit Fitness Center. For these IBC employees, informed consent will have been obtained (online) in advance of the in-person weight measurements. Specific language is outlined in the consent form reflecting this method of in-person weight measurements for IBC employees. Individuals weight data will be transferred to the research team via SecureShare; a web-based application used by the University of Pennsylvania for secure file exchange

Recruitment at SEPTA site:

After initial outreach by SEPTA’s communication channels, individuals will be asked go to the Way to Health (WTH) website for prescreening and eligibility ascertainment. In-person weight measurements will then be obtained at the SEPTA medical department by the research study staff, and also by the SEPTA nurse. The SEPTA nurse is also the main point-of-contact working with the research team for this study. The SEPTA nurse will obtain in-person weight measurements only on an as needed basis in the event research staff is not available to be on-site.

In-person weight measurements at the City of Philadelphia (CoP) site:

After initial outreach by the City of Philadelphia’s communication channels, individuals will be asked go to the Way to Health (WTH) website for prescreening and eligibility ascertainment. In-person weight measurements will then be obtained at the CoP site by the research study staff.

9.4 Subject Compensation

To enhance retention, we will provide participants a total of $250 for completing follow-up visits. Participants will receive $50 for completing weights at the screening baseline visit and at follow-up visits at 6, 12, 18, and 24 months regardless of weight. This strategy has minimized differential drop out in our previous studies. (For the lottery incentive payments, please refer to the 'Design of the Incentives' header in the Procedures section of this application)

10. Study Procedures

Consent Process

10.1 Overview

Upon the initial outreach, individuals who are interested in learning more about the study will be directed to the Way to Health web portal. Upon reaching the portal, participants will complete a screening intake form to determine their eligibility. If participants are interested in participating, the Way to Health portal will take them through an automated online written informed consent session. The consent session will be divided into sections and potential participants will have to click a button to advance through each section. This is to help ensure that participants read the consent form thoroughly by breaking down the form into manageable blocks of text. Each section will have a button allowing the user to contact a researcher via email if she has questions about the consent form. Successive screens will explain the voluntary nature of the study, the risks and benefits of participation, alternatives to participation, and that participants can cease to participate in the study at any time. On the final consent screen, potential participants who click a clearly delineated button stating that they agree to participate in the study will be considered to have consented to enroll. Participants will then be randomized to one of the four arms by the web application. The participant will be led through an automated description of the details specific to that arm. After receiving such information, participants

will be asked to confirm that they understand and wish to continue. Participants will be able to contact the research staff if they have any questions about their assigned condition before they proceed to the next step. Participants will be provided with details regarding how to contact the research team via email or phone at any time if they subsequently wish to withdraw from the study. This contact information will remain easily accessible via the subjects individual Way to Health web portal dashboards throughout the study.

Procedures

10.2 Baseline pre-treatment assessment for patients

Individuals who meet study entry criteria and have provided consent online will complete an intake survey measuring their health history, dietary intake, exercise habits, and previous weight loss efforts, and selected psychosocial variables shown to predict weight loss participation and success.

10.3 Randomization

Randomization: The study statistician will randomize study participants at study inception using a random number generator and permuted block randomization with variable block sizes to force balance among treatment group assignments; variable block sizes will preclude prediction of treatment assignments by study staff. The statistician will randomize in a 1:1 ratio for each intervention compared to control and will stratify by: (1) the employer site; (2) sex, and (3) degree of obesity at baseline (BM 30-37.9 and 38-55 kg/m2), as baseline BMI may moderate treatment effects. Following randomization, the study coordinator will provide instructions about the control, incentive, or environmental arms.

10.4 Environmental Change Strategies

The environmental change strategy arm will involve a menu of promising or evidence-based environmental change strategies to promote healthy eating and physical activity, delivered through print, website-based and mobile communication channels to employees assigned to the Environmental and Combined arms of the trial. The strategies have been identified through systematic literature reviews, through Dr. Glanz' previous intervention studies, and from discussions with collaborators working on novel environmental change strategies. The planned environmental change strategies are based on our preliminary assessment and discussions with the participating worksite informants during the development of this proposal and are circumscribed by the context of these urban worksites and the structure of their employees work activities. The interventions are targeted at individual employees rather than work groups, due to the inclusion criteria (i.e., obese employees with at least one other CVD risk factor), feasibility considerations (individual rather than group randomization, total resources available), and the potential for translation, scalability and dissemination. As in many urban worksites, there are no in- house cafeterias but employees have access to lunchrooms for limited food and beverage preparation and storage (refrigerators, microwave ovens, vending machines) and are located near numerous food sources (restaurants, quick-serve or fast-food restaurants, lunch trucks, snack bars). Also, these worksites do not have fitness facilities on-site but have ample access to

stairways, neighborhood gyms, and walking routes and parks within a 5-10 minute walk of their worksites.

Healthy eating environmental change strategies will be designed to guide participants in identifying environmental influences on excess food intake and making environmental modifications easier for them to implement in their worksites, in the area around worksites, and in their homes. At the worksite, the strategies will emphasize identifying healthy vending options, healthy snack access, social environment change, and establishing healthy catering policies for work-based events. In the neighborhood near the worksite, strategies will include tools to help workers find healthy choices easily at restaurants and periodic promotions (arranged with vendors) to encourage trying new healthful prepared foods. Home environment changes will be guided by the Home Food and Activity Environment Audit tool, originally developed for research conducted by Dr. Glanz and colleagues at the Emory Prevention Research Center and recently adapted for an urban, low-income community intervention. Physical activity environment change strategies will also include strategies for worksites, near worksites, and in homes. At the worksites, strategies will include individually-delivered prompts to use stairs, standing desks, and walking breaks. In the area near worksites, strategies will include guidance on safe, convenient walking routes, link to benefits for incentives toward gym memberships, and safe bicycling paths. The Home Food and Activity Environment Audit will address physical activity environments in homes. Environmental strategies will be communicated to participants in the Environmental and Combined study arms twice weekly during the first 6 months, and weekly during months 7-18.

They will be varied to maintain interest and offer a variety of approaches that participants can use. The Way to Health platform will be programmed to send links to web and mobile intervention information, and to prompt participants who wish to have hard copy/paper copies of intervention materials. One week after a strategy tool is distributed, a simple log will be provided for participants to indicate their liking for, and use of, the various strategies. All participants in each of the three intervention arms will also receive a free wireless scale (value $110) as this will be used to both obtain weights and provide participants with feedback. We are not providing these scales to participants in the usual care arm because that would not be consistent with usual care. Usual care: Employees of IBC, SEPTA, and City of Philadelphia are all offered the same wellness program that consists of: (1) yearly biometric screenings for which members receive incentives to participate; (2) reimbursements for fitness and weight management program participation (up to $150 per year for documentation of 120 workouts and up to $150 for documentation of participation in Weight Watchers); (3) Eat Right for Life educational program which includes onsite seminar and workshop materials; (4) six visits with a registered dietician as part of health benefit; (5) free use of WebMD tools - members biometric data is uploaded or self-reported and tools are available to the member to set goals and track progress.

Psychological research has identified social support as a factor that can help support weight loss. In an effort to strengthen the social support environment for participants in the environmental strategies and the combined incentives and environmental strategies arms, we will create a “secret” Facebook group where participants in these arms will be encouraged to provide support and seek support from one another. We will encourage wall posts of text, healthy recipes, pictures related to nutrition, physical

activity and the environment. The research study team will periodically post helpful links or tips that participants can use to improve their nutrition and physical activity environment.

Language added to the latest version of the informed consent document states that we will ask participants assigned to some arms (who wish to join a voluntary Facebook group) to provide us with their Facebook e-mail address. Participants will be asked to provide us with their Facebook e-mail address by completing a single question survey on their personal account page on the Way to Health platform. Preceding the survey will be a Facebook addendum to the consent explaining more in depth details of participation in the Facebook group. Participants will be informed here that they can view and print a version of the Facebook addendum from their personal Healthy Weigh account page at any time. This form will also direct participants to view the "study instructions" section of their dashboard content for more specific information on how to locate one's Facebook e-mail address, how to create a Facebook account, and how to accept our Facebook invitation. If participants do not have a Facebook profile, or choose not to provide us with this information, they may still participate in the study. This will be notated in the Way to Health dashboard content and on the Facebook addendum. We will invite participants in the environmental strategies arms to join the group called "Healthy Weigh," via the Facebook e-mail address that they provide through the single question survey. Participant identity will only be revealed to other enrolled subjects in the environmental strategies arms if a participant decides to join the Facebook group. If a participant declines, they will remain anonymous.

The privacy type for the group will be "Secret." This means that only research staff will have control over who joins the group. Only current or former group members, people who were in the group but left, can see the group's name. No one outside of the study will be able to locate, view or join the group without an invitation from the study team. Only current members can see who else is in the group. Only current and former members can see group activity. Only current members can post in the group. Only current and former members will be able to find the group in a search. Only current members can see stories about the group on Facebook (in the News Feed and search).

The research study team will closely monitor Facebook group activity in order to ensure that all activity occurring with the group is appropriate and related to the study. If an inappropriate post is made the research study team will promptly remove the post, and will contact the participant who made the post to review the purpose of the group and to review with the participant which types of posts are appropriate for the purposes of the research study. Inappropriate posts are any posts that do not relate to weight loss, social support, nutrition, physical activity, or the environment.

We intend to analyze the Facebook activity of participants within the Facebook group in order to determine: how many and which participants opt to join the group; how many posts, likes, and comments each participant makes during the study period; and whether the amount or nature of Facebook group activity corresponds with weight loss success. We also plan to look qualitatively at the nature of the posts, likes and comments of participants. No data analysis will be conducted until the end of the intervention. Data will be transcribed directly from Facebook into an excel sheet for tracking and organization of participant activity. This task will be completed by the research study personnel. The excel sheet will be stored in a password protected file on the secure/firewall servers of the PMACS

Data Center, in data files that will be protected by multiple password layers. These data servers are maintained in a guarded facility behind several locked doors, with very limited physical access rights. An additional amendment will be forthcoming, which will explain the detailed data analysis process.

Outcome assessments: Weight for all participants will be ascertained on enrollment in the study and then at the end of Phase I (6 months), at 12 and 18 months (end of Phase II), and at 24 months (end of Phase III). The primary outcome will be change in weight from baseline to 18 months. We will utilize other measures collected at months 6, 12, 18 and 24 as part of routine biometric assessments by the participants' employers, all of whom share the same insurance carrier, Independence Blue Cross (IBC).

10.5 Design of the Incentives

The incentive programs incorporate key aspects of optimal design, including objective and reliable confirmation of behavior change at specified intervals (weight measurement), reinforcement of the target behavior (weight maintenance) with non-drug reinforcers (incentives), large payments to reinforce the target behavior, and intermittent reinforcement. All incentive participants will receive daily feedback for 18 months; they will be given an electronic scale (accurate to ±0.2 lbs) and given a weight loss goal for the first 6 months of 0.5 lb a week. At the beginning of Phase II and each month from months 7-18 they will be asked to set a goal of either maintaining weight loss or losing further weight (e.g. choice will be either 0, 0.5 pounds per week, with a default of 0 pounds if nothing is chosen). Inviting people to set more ambitious goals than maintenance is based on evidence that people often voluntarily commit to more ambitious goals than would be economically rational and this commitment can help them achieve those goals.

Each month we will provided each study participant with a graph on the web portal that shows a line beginning at their current weight and dropping to their target weight over the remaining months of Phase I (see Figure 4 in grant proposal, which presents a sample weight loss line for a participant who begins Phase I at 250 lbs and sets a one pound per week weight loss goal over 6 months ideal weight loss trajectory and fresh start trajectory described below.). Participants will self-monitor weights daily. Each morning before eating or drinking and after urinating, participants will weigh themselves and their weights will automatically be transmitted by the Withings Scales to the Way to Health system.

Participants will then receive automated feedback from the system (they may choose text or email) on their progress relative to their goals and their potential earnings, helping to make the payments feel immediate. They will be told that they will receive the full amount if they meet the 6 month target for their weight (the sum of the monthly weight loss goals) but half the money if they do not. Daily monitoring provides participants with a frequent reminder of their goals, chosen because people tend to adopt a narrow frame of reference to diets, rededicating themselves each day. Daily weigh-ins should

reduce binge eating by making participants constantly accountable for their weight. Monthly graphical feedback provides encouragement and a symbolic reward for slow and steady progress. Participants in the lottery arm will be eligible for a daily lottery prize with an expected value of $3.00/day if, prior to resolution of the lottery, they reported a weight at or below their goal on the ideal weight loss trajectory in Figure 4. The lottery provides both infrequent large payoffs (a 1 in 100 chance at a $100 reward) and frequent small payoffs (a 1 in 5 chance at a $10 reward) since subjects are motivated by both the future (fixation on large potential winnings) and the past (how often did I win?). Subjects will be assigned a 2-digit number with two distinct digits upon entry into the study, e.g. 27. A two digit random number will be generated for each day. If the last digit includes either a 2 or 7" (a 1 in 5 chance), and the subject reported a weight at or below target weight, s/he wins $10. If the two digits are 27 (a 1 in 100 chance) the subject wins an additional $100. When a subjet reports a weight above target, we will still generate a 2- digit daily number and tell the subject if s/he would have won the lottery that day if s/he had reported a weight at or below target. We believe that a desire to avoid the regret associated with not winning combined with learning that one would have won, had one been adherent, will motivate participants to a greater degree than only on the value of the rewards. A key aspect of the weight loss trajectory is that it can be reset monthly, a critical feature in the likely event that a participant falls short of attaining a monthly weight loss goal. To avoid discouraging such subjects, who would otherwise suddenly have to lose a lot of weight to get back on track and might decide to drop out of the study, each participant will be given the opportunity for a fresh start. The overall goals set at beginning of Phase I will stay the same, but the slope of the trajectory will adjust (i.e., steepen) such that the participant need not binge diet following a month of poor weight loss performance (see Fresh start trajectory in Figure 3, for a subject who lost 1 lb. instead of 4 lbs. in first month). Keeping the overall weight loss goal constant makes the procedure fair for participants who maintain the ideal trajectory, while adjusting the slope of the weight loss line allows for recovery from slip-ups, an approach we have used to attain minimal lost to follow-up rates of less than 10% in previous weight loss studies. To give participants frequent feedback, we will pay out 50% of winnings to date at the end of each month. To minimize falsification of weights and to utilize the amplification of motivation provided by loss aversion, the other half will be held in a virtual account and paid to participants at the end of each 6 month period only if they have met or exceeded their monthly goal. Between months 19-24 (Phase III), participants will not receive incentives tied to weight maintenance to evaluate sustainability of effects achieved in Phases I and II.

10.6 Study Duration

The duration of participation for each individual participant is expected to be is 2 years (24 months). We plan to recruit and enroll participants starting in April 2015, until June 2015 (over a three month time period). We expect the total duration of the study to last 5 years (60 months) for the completion of all three phases of the study.

10.7 Dissemination

Findings from this research will be disseminated through incorporation into the wellness/benefits design for employees of City of Philadelphia, SEPTA, and IBC. Broader dissemination will include use in benefit

design approaches used for IBC throughout the region and more widely through collaborating organizations nationally, other employers, and health delivery organizations once findings are published.

11. Analysis Plan

11.1 Statistical Considerations

Descriptive analyses: We will produce data summaries regularly using frequencies for categorical variables and means, medians, and ranges for continuous variables. We will assess data quality and examine distributional assumptions with graphical methods. To evaluate balance among groups achieved by randomization, we will compare baseline values of all variables across the 4 arms using appropriate tests. General procedures: All primary analyses will be on an intent-to-treat basis including each participant in the group to which s/he was randomized, regardless of adherence to the assigned strategy. Given the high rates of missing data typical of weight loss studies, handling of missing data is an important issue. Primary analyses will assume that any participants for whom follow-up weight loss data are unavailable have had their weight return to baseline (weight at beginning of Pre Phase). Within each arm, this assumption is likely to be conservative but this may not be the case in inter-arm comparisons, depending on the dropout rates in the different arms and true follow-up weights of the dropouts. Thus, a key secondary analysis will consider the effect of differential dropout among the treatment arms. Specific analysis plans are described below. All hypotheses will be tested using two-sided, 0.05-level tests unless otherwise specified (notably the Tukey honest significant difference testing approach will used for the three primary hypotheses in Specific Aims 1-3). Specific contrasts: Specific Aim 1. Assess the effectiveness of a daily lottery-based financial incentive, relative to the control group, on cumulative weight loss over an 18-month period. H1: Mean weight loss from baseline to 18 months among participants randomized to the lottery incentive will be at least 5 kg greater than among control group participants. The primary analyses will be unadjusted intent-to-treat analyses, using a t-test for differences between each intervention group and the control group in weight change between baseline and 18 months, applying the Tukey honest significant difference testing procedure. If weight change appears to be non-normally distributed, we will find an appropriate transformation or use the non-parametric Wilcoxon rank-sum test. Missing data will be handled by assuming that participants who are lost to follow-up have returned to their initial weight, i.e., using baseline weights for imputation. Weight change between baseline and 18 months, with 95% confidence intervals (CIs), will be estimated for comparison with adjusted analyses. We will also estimate regression models adjusted for the stratification variables (sex, employer, initial BMI) and other demographic factors including race, income, and education. If there is evidence of confounding using change in estimate criterion, we will use multivariable linear regression models to compute adjusted estimates of difference in weight change between 0 and 18 months between the arms, with 95% CIs. We will fit exploratory models of the repeated weight measurements that incorporate time as a polynomial function or using visit-specific indicator variables to determine the most parsimonious model that adequately describes the observed patterns. We will investigate random-effects models that allow for baseline individual variability as well as variability in the changes in weights over time; an example is the following: E(weightij) = 0 +1Time j + 2Groupi + 3 Xi + b0i + b1i Time j , where i indicates subject, j indicates assessment times, the

parameters are fixed effects linking time, a treatment group vector, and a vector of other demographic or clinical covariates X i to the outcomes, and b0i and b1i are random intercept and slope effects. Tests for significance of random effects will use likelihood ratio tests for nested models; we will compare models with different random effects structures using the maximized log-likelihoods and the Akaike Information Criterion (AIC). We will apply standard diagnostic techniques to assess model adequacy. We will use treatment by time interactions to assess whether the rate of change in weight differs by intervention arm. We hypothesize that incentives may be more effective among lower income individuals and will evaluate this using interaction terms of income with treatment group. We will explore differential effects by race and education and baseline levels of intrinsic motivation and stages of change. To assess the sensitivity of treatment effect estimates to missing data, we will fit hierarchical or mixed effects models with and without accounting for informative missing data. Specific Aim 2: Assess the effectiveness of environmental strategies, relative to the control group, on cumulative weight loss over an 18-month period. H2: Mean weight loss from baseline to 18 months among participants randomized to the environmental strategies group will be at least 5 kg greater than among control group participants The analysis will proceed similar to the analysis of Specific Aim 1. Specific Aim 3. Assess the effectiveness of the combined incentives and environmental strategies arm, relative to the control group, on cumulative weight loss over an 18-month period. H3: Mean weight loss from baseline to 18 months among participants randomized to the combined arm will be at least 8 kg greater than among control group participants. The analysis will proceed similar to the analysis of Specific Aims 1 and 2. Specific Aim 4. Assess the comparative effectiveness of lottery-based incentives and environmental strategies on cumulative weight loss over an 18-month period. H4: Mean weight loss among participants randomized to the combined arm will be at least 3 kg greater than among participants in either of the individual active arms. The analysis will proceed similarly to the analysis of Specific Aims 1-3 Specific Aim 5: Assess the costs of each of the intervention arms from both the employer and social perspective and compare the cost differences between each arm relative to the effectiveness measured by incremental weight loss achieved. H5: From an employer's perspective, all intervention arms will be cost-effective relative to the control group but the combined arm will be the most cost effective. The principal incremental cost-effectiveness ratios between intervention and control arms will be from the employers perspective and compare costs during the intervention from baseline to 18 months per unit change in weight. Secondary analyses will 1) evaluate this same ratio but use either 6 months post-intervention data (24 month visit) or 12 months of intervention data (12 month visit) and 2) evaluate these 2 ratios using a limited social perspective. Cost models will use a generalized linear model with a log link and gamma family. Missing data strategies will parallel those described above. We will assess sampling uncertainty for the comparison of costs and effects by calculating parametric 95% CIs for the cost per kg lost and acceptability curves. Standard errors and correlation of the difference in cost and effect will be derived using a bootstrap procedure.

11.2 Power and Sample Size Considerations

We have designed the study with adequate power to detect differences in weight loss over an 18-month period. Our intervention should achieve its maximal impact in maintaining initial weight loss at the end of Phase II (month 18), when the incentive payments cease. To maintain the experiment-wide Type I

error and guard against false conclusions of effectiveness, we use the Tukey honest significant difference approach which maintains power to show significance of at least one of the three primary comparisons (each intervention relative to control) and at least one of the secondary comparisons (the combined group relative to each of the two interventions alone). If the interventions are as effective as expected, we will have 90% power to detect the improvement in our primary outcome in at least one intervention group compared to control. We plan to recruit approximately one third of the 328 participants required for the start of Phase I from each of our participating employers (IBC, SEPTA, and City of Philadelphia). Given the large number of potentially eligible employees, we can, if needed, easily increase the proportion of participants from any of these employers to meet recruitment targets. The 328 participants will be randomized to our 3 intervention groups and the control group using a 1:1 ratio for the intervention arms. We have built in a margin of 20% for potential attrition before the 18- month assessment, resulting in 260 participants (65 per arm) who are available for analysis at the end of Phase II. This will provide us with 90% power to detect a difference in weight change between baseline and the 18-month weigh-in of 5 kg between each intervention group and the control group and 3 kg between the combined group and either the incentive or environmental strategies groups. Sample size/ power analysis. Primary outcomes. Specific Aims 1-3: Compare effectiveness of financial incentive, environmental strategy, and combined arm to control during Phases I and II For expected weight change between months 0-18 of -8 kg, -5 kg and 0 kg in the combined, intervention and control groups, respectively (a net difference between single intervention and control groups of 5 kg and between combined intervention and control groups of 8 kg) and assuming a standard deviation (S.D.) of weight loss of 5 kg, 65 participants per group provide 90% power to detect at least one of these contrasts. The sample size provides 80% power to detect a difference of 4.6 kg in weight change between single intervention and control groups and 7.3 kg between the combined and control groups. Specific Aim 4: Compare effectiveness of financial incentives and environmental strategies and combined arm during Phases I and II This test is powered like Specific Aims 1-3 but designed to detect a smaller difference, taking advantage of the Tukey approach; the sample size of 65 per arm provides 90% power to detect a difference of 3 kg and 80% power to detect a difference of 2.7 kg. Secondary outcomes. Specific Aim 5: Compare the cost differences between each arm relative to the effectiveness measured by incremental weight loss achieved. We will calculate a point estimate of cost per kg of weight loss based on estimated inter-arm differences in weight loss and the incremental cost of the interventions, as described in C.3.d.ii.c. For weight loss at 18 months, given an expected incremental cost of $810 (incentives) and incremental weight loss of 5 kg, 328 participants provide 80% power to detect a cost per kg lost of $430 and greater than 90% power to detect a cost per kg lost of $575. This study is primarily a test of the efficacy of these interventions. Due to resource constraints and because we do not yet know about intervention effectiveness, we did not power this study based on cost effectiveness analyses. Estimated detectable costs per kg are a function of wide confidence intervals due to sample size.

11.3 Measurement of effect:

The goal of this analysis will be to assess relative weight loss in the intervention arms relative to control. For the principal cost-effectiveness analysis, measurement of incremental weight loss will be based

on differences in weight between baseline and the 18 month visit; in secondary analyses it will be based on weight differences between baseline and 24 months and baseline and 12 months.

12. Human Research Protection

12.1 Subject Confidentiality

Each participant will be assigned a unique, numeric identifier which will be used on all collected study information. The source document in which the unique identifier is associated with personal information will be stored in a password protected computer file to which only study personnel have access. All data will be stored on the Way to Health web-based platform database. Threats to confidentiality will be minimized by careful data collection and the private and secure web-based platform. At the conclusion of the study, all identifying information will be destroyed and all data will be archived in a password protected folder. The web application for this study will use account-based authentication and permission systems to protect confidentiality. An investigator or statistician who logs in will be able to access only de-identified data. Only research staff will be responsible for contacting participants for study related activities (responding to questions about the study) will be able to view participant names and contact information. All of these personnel will have completed research and confidentiality (CITI) training. The system will automatically generate logs of all data queries, and these will be reviewed weekly by research staff to ensure that no unauthorized persons have gained access to identifiable information. We will implement multiple, redundant protective measures to guarantee the privacy and security of the participant data. All investigators and research staff with direct access to the identifiable data will be required to undergo annual responsible conduct of research, cybersecurity, and Health Insurance Portability and Accountability Act certification in accordance with University of Pennsylvania regulations. All data for this project will be stored on the secure/firewalled servers of the PMACS Data Center, in data files that will be protected by multiple password layers. These data servers are maintained in a guarded facility behind several locked doors, with very limited physical access rights. They are also cyber-protected by extensive firewalls and multiple layers of communication encryption. Electronic access rights are carefully controlled by University of Pennsylvania system managers. We will use highly secure methods of data encryption for all transactions involving participants financial information using a level of security comparable to what is used in commercial financial transactions. We believe this multi-layer system of data security, identical to the system protecting the University of Pennsylvania Health Systems medical records, greatly minimizes the risk of loss of privacy. All study-related emails will be sent to non-work based email addresses. Research material will be obtained from participant surveys, and the wireless scales. All participants will provide informed consent for access to these materials. Research material that is obtained will be used for research purposes only. The same procedure used for the analysis of automated data sources to ensure protection of individuals' information will be used for the survey data, in that individual identifiers will be used only for linkage purposes or to contact individuals. The study identification number, and not other identifying information, will be used on all data collection and contained in these databases. All study staff will be reminded to appreciate the confidential nature of the data collected and contained in these databases. The UPenn Biomedical Informatics Consortium (BMIC) will be the hub for the hardware and database infrastructure that will support the project and where the Way to Health web

portal is based. The BMIC is a joint effort of the University of Pennsylvania's Abramson Cancer Center, the Cardiovascular Institute, the Department of Pathology, and the Leonard Davis Institute.

12.2 Subject Privacy

Privacy refers to the person's desire to control access of others to themselves. Privacy concerns people, whereas confidentiality concerns data. Describe the strategies to protect privacy giving consideration to the following: The degree to which privacy can be expected in the proposed research and the safeguards that will be put into place to respect those boundaries. The methods used to identify and contact potential participants. The settings in which an individual will be interacting with an investigator. The privacy guidelines developed by relevant professions, professional associations and scholarly disciplines (e.g., psychiatry, genetic counseling, oral history, anthropology, psychology).

With assistance from IBC, SEPTA, and the City of Philadelphia, we will advertise this as a research study of different ways to help people lose and keep off weight. The initial recruitment outreach will be facilitated by IBC, SEPTA, and the City of Philadelphia, utilizing existing communication channels (as described in the recruitment section). Interested participants will be asked to go to the Way to Health (WTH) website for prescreening and eligibility ascertainment. Screening intake will be done using the WTH website supplemented by phone support as needed, with the final step being confirmation of weight by validated employee biometric assessment data (within 4 weeks), or through participants physicians (documentation provided from the patient's outreach to their physician). During the screening intake on the WTH website, we will collect subjects names, addresses, email addresses, phone numbers, height and weight, medical history, sociodemographic data, social security numbers, and employees' biometric assessment data. All of these data will be stored in an encrypted database that conforms to applicable data security standards. Access to all such data will be limited to specifically designated research staff that will be responsible for contacting participants for follow-ups and responding to questions and concerns from participants. All communications between participants and the study website will be encrypted with SSL/HTTPs technology. Social security numbers, bank account numbers and routing numbers for all persons to whom rewards are sent will be transmitted in encrypted format to Accounts Payable, who will store the data for W-9 forms. After the social security numbers are no longer needed they will be deleted from our system.

12.3 Protection of Human Participants:

Human subjects involvement and characteristics. For the proposed study, 328 subjects with a BMI of 30 to 55 kg/m2 and over the age of 21 who are full- time employees of SEPTA, IBC and the City of Philadelphia will be eligible for study. Eligibility will be determined from the study inclusion/exclusion criteria (Refer to inclusion/exclusion section of the protocol application). Exclusion criteria were limited to factors that may confound results or that make participation in a weight loss program infeasible, unsafe, or require more intensive monitoring. These exclusions include unstable heart disease, uncontrolled hypertension, kidney disease, and other serious chronic illness (e.g., transplant recipient, terminal illness). Patients with diabetes mellitus on hypoglycemic medication other than metformin will be excluded because medication adjustments and monitoring that they would require is beyond the

scope of this trial. Patients on diabetes or high dose diuretic medication are excluded because these medications may require adjustment during weight loss to prevent hypoglycemia and dehydration. Women who are pregnant or breastfeeding are excluded because their energy requirements are obviously different from other individuals. Substance abuse and unstable mental illness were chosen because adherence to the intervention may be disproportionately difficult in these individuals compared with other individuals. We will exclude temps or part-time employees because lost to follow-up rates over 24 months may be high. Persons with a history of bulimia nervosa-related behaviors are excluded because they may employ these behaviors to reach their weight goals. We will also exclude potential subjects who cannot or will not give consent. No subjects will be excluded on the basis of sex or race. There will be approximately equal representation of African-Americans and Caucasians and 30%-40% men based on our recruitment experience in previous weight loss clinical trials and the demographics of the employer populations.

12.4 Cost-Effectiveness Analysis.

Participants will be asked to provide informed consent when enrolled in the research project after full explanation of the research project. For the cost- effectiveness analyses, we will not be using claims data so HIPAA authorization related to access to their medical charts will not be required. We will request access to all biometric data collected as part of employer wellness programs that study participants are part of. All participants will be over the age of 21. Women and minorities will be included.

12.5 Sources of materials.

All research material obtained from study participants will be gathered prospectively and will include weight, and subjects responses to questionnaires. We will obtain biometric information through the participants employer as this information is already collected as part of the employee wellness program. In addition, research material that is obtained will be used for research purposes only.

12.6 Potential Risks Involved in the Proposed Study.

There are minimal risks associated with providing lottery-based financial incentives and making environmental manipulations to encourage initial and maintenance of weight loss. The main risk is loss of confidentiality, which will be protected as described below. There are no potential risks associated with any other measures or data to be collected. During the consent process, we will inform subjects of the risks associated with loss of confidentiality.

12.7 Risks Involved in the Cost-Effectiveness Analysis.

As stated above, the main risk is loss of confidentiality. The immediate benefits of this study for participants are minimal; however, as mentioned, so are the risks. Overall the risk to benefit ratio is highly favorable given the long term potential of this study to significantly contribute to our knowledge

of financial incentive and environmental strategies to combat obesity and their impact on health and health-related behaviors.

12.8 Adequacy of Protection from Risks Recruitment and informed consent.

With assistance from IBC, SEPTA, and the City Department of Public Health we will advertise this study as a research study of different ways to help people lose and keep off weight. Potential participants will be told that some participants will also receive financial incentives, but this feature will not be advertised, lest subjects enlist primarily for that reason. Interested participants will be asked to go to the Way to Health (WTH) website or to contact study personnel by phone for prescreening and eligibility ascertainment. Intake will be done using the WTH website supplemented by phone support as needed. Enrollment will include a description of the voluntary nature of participation, the study procedures, risks and potential benefits in detail. The enrollment procedure will provide the opportunity for potential participants to ask questions and review the consent form information with family and friends prior to making a decision to participate. Participants will be told that they do not have to answer any questions if they do not wish and can drop out of the study at any time, without affecting their medical care or the cost of their care. They will be told that they may not benefit directly from the study and that all information will be kept strictly confidential, except as required by law.

12.9 Protection against risk.

In the event of an adverse effect necessitating medical or professional intervention, referral to the study participants primary care provider or an appropriate specialist will occur. In emergency situations when a study participant contacts one of the research personnel first, the particular research staff member will either make contact with emergency personnel or advise the study participant to do so immediately and follow up with the emergency personnel to ensure communication of the study interventions and their associated risks. Participants will be queried for potentially relevant health status changes at each data collection visit throughout follow-up. Additionally, an independent DSMB will review the clinical data routinely with safety as its primary objective. No results will be reported in a personally identifiable manner.

12.10 Electronic Data Security.

The same procedure used for the analysis of automated data sources to ensure protection of patient information will be used for the survey data, in that patient identifiers will be used only for linkage purposes or to contact patients. The study identification number, and not other identifying information, will be used on all data collection instruments. All study staff will be reminded to appreciate the confidential nature of the data collected and contained in these databases. The Penn Medicine Academic Computing Services (PMACS) will be the hub for the hardware and database infrastructure that will support the project and where the Way to Health web portal is based. PMACS provides a secure computing environment for a large volume of highly sensitive data, including clinical, genetic, socioeconomic, and financial information. Among the IT projects currently managed by PMACS are: (1) the capture and organization of complex, longitudinal clinical data via web and clinical applications portals from cancer patients enrolled in clinical trials; (2) the integration of genetic array databases and

clinical data obtained from patients with cardiovascular disease; (3) computational biology and cytometry database management and analyses; (4) economic and health policy research using Medicare claims from over 40 million Medicare beneficiaries. PMACS requires all users of data or applications on PMAC servers to complete a PMACS-hosted cybersecurity awareness course annually, which stresses federal data security policies under data use agreements with the university. Curriculum includes HIPAA training and covers secure data transfer, passwords, computer security habits and knowledge of what constitutes misuse or inappropriate use of the server. We will implement multiple, redundant protective measures to guarantee the privacy and security of the participant data. All investigators and research staff with direct access to the identifiable data will be required to undergo annual responsible conduct of research, cybersecurity, and Health Insurance Portability and Accountability Act certification in accordance with University of Pennsylvania regulations. All data for this project will be stored on the secure/firewalled servers of the PMACS Data Center, in data files that will be protected by multiple password layers. These data servers are maintained in a guarded facility behind several locked doors, with very limited physical access rights. They are also cyber-protected by extensive firewalls and multiple layers of communication encryption. Electronic access rights are carefully controlled by University of Pennsylvania system managers. We will use highly secure methods of data encryption for all transactions involving participants financial information using a level of security comparable to what is used in commercial financial transactions. We believe this multi-layer system of data security, identical to the system protecting the University of Pennsylvania Health Systems medical records, greatly minimizes the risk of loss of privacy.

12.11 Potential Study Benefits

Potential Benefits of the Proposed Research to the Participants and Others Participants in this study will benefit directly by receiving free health monitoring as well as weight management, nutrition, and physical activity education. The benefits of weight loss are many; weight loss can result in improvements in hypertension, hyperglycemia, dyslipidemia, arthritis, sleep apnea, and many other obesity-associated conditions. More importantly, while the subjects themselves might not derive benefit, knowledge gained from the study will assist in the treatment of others who are overweight or who attempt weight loss by one of the methods studied.

12.12 Resources necessary for Human Research Protection

The proposed research project will be conducted by a team based within an environment at the University of Pennsylvania (UPENN) that provides substantial research experience, infrastructure support, and expertise in areas important to this project. In particular, the Center for Health Incentives and Behavioral Economics (LDI CHIBE) is one of 2 NIH-funded Centers in Behavioral Economics and Health in the United States and has developed an NIH-funded infrastructure (the Way to Health) for conducting behavioral interventions using a combination of wireless technologies and automated feedback including participant payment of incentives and the Center for Health Behavior Research (CHBR) has extensive community based research experience including work on environmental change strategies. The team includes investigators experienced in obesity interventions, clinical trials, health economics research, behavioral economics, and cost-effectiveness analyses. Dr. Kevin Volpp (Multiple

PI) is an expert in behavioral economics and health and directs the Center for Health Incentives and Behavioral Economics (CHIBE) and the NIA-funded Penn CMU Roybal P30 Center on Behavioral Economics and Health. He is a Professor of Medicine at the Perelman School of Medicine and Professor of Health Care Management at the Wharton School as well as an elected member of the Institute of Medicine of the National Academy of Sciences. Dr. Karen Glanz (Multiple PI) is an expert in the field of obesity research, nutrition, and physical activity behavior and interventions and is the George A. Weiss University Professor of Epidemiology and Nursing at the University of Pennsylvania and also an elected member of the Institute of Medicine; Dr. Pamela Shaw, a biostatistician experienced in the design and analysis of weight loss intervention studies and an Assistant Professor of Biostatistics at UPENN, will lead the statistical analyses; Dr. Will Yancy (consultant) is an expert in the design and conduct of clinical trials of weight loss interventions and Associate Professor of Medicine at Duke University School of Medicine. Dr. George Loewenstein (Consultant) is the Herbert A. Simon Professor of Economics and Psychology at Carnegie Mellon University and a founder of the fields of behavioral economics and neuroeconomics who has led or co- led both incentive and environmental interventions to reduce obesity. We have successfully completed 4 weight loss RCTs using financial incentive interventions as well as worksite and community intervention studies using environmental strategies. We plan to enroll employees from the Southeastern Pennsylvania Transportation Authority (SEPTA), Independence Blue Cross (IBC), and the City of Philadelphia. There are an estimated 4,800 employees from these entities who have BMI greater than 30 and 1 or more cardiovascular risk factor, of whom approximately 48% are black.174 We plan to enroll 328 participants over 6 months making our recruitment targets easily attainable given the size of the recruitment pool. We have extensive experience working with outside entities (Weight Watchers, CVS Caremark, Humana, Horizon Blue Cross Blue Shield, Aramark are a few examples) and have enrolled thousands of participants in behavioral economic interventions around the United States and always achieved our enrollment targets. All senior/key personnel and research staff who will be involved in the design and conduct of the trial have completed the mandated human subjects research certificate program. In addition, all staff working on this study are required to carefully read the protocol and will be trained on all related study procedures and information. The multiple PIs will be responsible for ensuring that project faculty and staff have the equipment and training required to protect privacy and confidentiality and will monitor and document that these individuals are properly certified. If new senior/key personnel and staff become involved in the research, documentation that they have received the required education will be included in the annual progress reports.

13. Data Protection

13.1 Data and Safety Monitoring

Individual-level data for participants will be kept confidential and will only be stored on highly secure servers. Only authorized project personnel will have access to the data and the data will be stored on servers only and not stand-alone PCs or laptops. All data will be reported at units of aggregation which make it impossible the identification of individual subjects. However, because we are contacting individuals after their initial enrollment, there is an obvious need to have data with identifiers and contact information from the master enrollment files. Study personnel who work with these data will

have undergone all of the required human subjects training. They will work with the data in password protected files. The data and safety monitoring plan will have three parts. 1) PMACS will develop and implement methods of verifying entered data and of quality control. 2) the PIs will be directly responsible for identifying and reporting all serious adverse events, protocol deviations/violations and unanticipated events to the IRBs and funding agency promptly, as appropriate. The PIs will also report all adverse events, accrual rates, retention rates, and all other logistical issues to the DSMB (described below) at least biannually (and more frequently if there are serious adverse events). 3) there will be a data safety monitoring board (DSMB) responsible for monitoring the trial. Specifically, the multiple principal investigators (PIs) and the IRB will be responsible for ensuring risks to human subjects are minimized, risks are reasonable, subject selection is equitable, the research team has access to adequate resources to conduct the study, the informed consent process meets regulatory and ethical requirements, adequate provision is made to protect human subjects by monitoring the data collected and there are adequate provisions to protect subject privacy per HIPAA regulations and confidentiality of data. All senior/key personnel and research staff who will be involved in the design and conduct of the study must receive education in human research subject protection from a training program that is approved by a properly constituted independent Ethics Committee or Institutional Review Board. The multiple PIs will be responsible for ensuring that project faculty and staff have the equipment and training required to protect privacy and confidentiality and will monitor and document that these individuals are properly certified. If new senior/key personnel and staff become involved in the research, documentation that they have received the required education will be included in the annual progress reports.

13.2 Data and Safety Monitoring Board

The DSMB will be composed of 3 experts, with expertise in clinical trials, obesity research, and biostatistics, along with multiple PIs Drs. Glanz and Volpp as non-voting members. We consider the proposed trial to be relatively low risk. Therefore, we plan to arrange for a monitoring committee that is assigned to review the study and staff training protocols, monitor the trial for safety and adverse events, and conduct a semi- annual meeting. These members will not be involved directly with the trial and will perform several duties. First, they will review and approve the research protocol and plans for data and safety monitoring prior to initiation of the study. Second, they will evaluate the progress of the trial. This will include assessment of data quality, participant recruitment, accrual and retention, participant risk versus benefit, performance of trial sites, and study outcomes. This assessment will be performed at meetings every 6 months during the clinical trial and more frequently if needed. Third, they will make recommendations to ensure that all of the issues above are appropriately addressed. The multiple PIs of the project will be responsible for responding to all recommendations of the DSMB and submitting DSMB reports to the respective IRBs. We will identify members for the DSMB when the project is funded.

13.3 Potential Study Benefits

Potential Benefits of the Proposed Research to the Participants and Others Participants in this study will benefit directly by receiving free health monitoring as well as weight management, nutrition, and

physical activity education. The benefits of weight loss are many; weight loss can result in improvements in hypertension, hyperglycemia, dyslipidemia, arthritis, sleep apnea, and many other obesity-associated conditions. More importantly, while the subjects themselves might not derive benefit, knowledge gained from the study will assist in the treatment of others who are overweight or who attempt weight loss by one of the methods studied.

13.4 Alternatives to Participation

Participants may always consult with their primary care physician regarding ways to lose weight.

13.5 Risk/Benefit Assessment

Anticipated risks of this study should be minimal and the risk/benefit ratio is extremely favorable. Participants who are obese are at greatly increased risk of many medical problems including diabetes, hypertension, cardiovascular disease, musculoskeletal disorders, and certain cancers. Losing weight would significantly lower these risks. To minimize the chance for serious and unexpected adverse events, study participants will be screened through exclusion criteria for any health conditions that may be exacerbated by participating in a weight loss study. Our incentives try to motivate a gradual rate of weight loss that should pose little health risk in participants with BMIs of at least 30 on enrollment. Weight loss will be monitored electronically and participants who are losing more than 5 pounds in a week, 8 pounds in two weeks or 12 pounds in four consecutive weeks will be asked to slow down the pace of their weight loss, and will be reminded that they do not receive any additional incentive for losing more than an average expected pounds per month.

Healthy Weigh Statistical Analysis Plan - Modification

Version 7/26/2018

Overall Objectives/Specific Aims

Primary

6. Assess the effectiveness of a daily lottery-based financial incentive, relative to the control group, on cumulative weight loss over an 18-month period. *H1: Mean weight loss from baseline to 18 months among participants randomized to the lottery incentive will be at least 5 kg greater than among control group participants.*

7. Assess the effectiveness of environmental strategies, relative to the control group, on cumulative weight loss over an 18-month period. *H2: Mean weight loss from baseline to 18 months among participants randomized to the environmental strategies group will be at least 5 kg greater than among control group participants.*

8. Assess the effectiveness of the combined incentives and environmental strategies arm, relative to the control group, on cumulative weight loss over an 18-month period. *H3: Mean weight loss from baseline to 18 months among participants randomized to the combined arm will be at least 8 kg greater than among control group participants.*

9. Assess the comparative effectiveness of a combination of lottery-based financial incentives and environmental strategies on cumulative weight loss over an 18-month period. *H4: Mean weight loss among participants randomized to the combined arm will be at least 3 kg greater than among participants in either of the individual arms.*

Secondary

10. Assess costs of each of the intervention arms from both the employer and social perspective and compare cost differences between each arm relative to effectiveness measured by incremental weight loss achieved. *H5: From an employer's perspective, each individual intervention will have favorable cost-effectiveness ratios relative to control; the combined arm will have a favorable cost-effectiveness ratio relative to individual arms.*

5. Primary Outcome Variable

5.1 Change in weight from baseline to 18 months.

5.2 Specific Aims 1-3

Compare effectiveness of financial incentive, environmental strategy, and combined arm to control during Phases I and II. For expected weight change between months 0-18 of -8 kg, -5 kg and 0 kg in the combined, intervention and control groups, respectively (a net difference between single intervention and control groups of 5 kg and between combined intervention and control groups of 8 kg) and assuming a standard deviation (SD) of weight loss of 5 kg, 65 participants per group provide greater than

90% power to detect a difference with each intervention arm and control. With this sample size, there will also be approximately 87% power to detect a difference between the combined intervention arm and each of the single intervention arms. These calculations assume a 20% missing rate at the end of Phase II and hypothesis testing done using the Holm p-value correction for multiple comparisons (more details provided below).

5.3 Specific Aim 4

Compare effectiveness of financial incentives and environmental strategies and combined arm during Phases I and II.

6.0 Secondary Outcome Variable(s)

Specific Aim 5

6.1 Compare the cost differences between each arm relative to the effectiveness measured by incremental weight loss achieved.

7.0 Study Design

8.1 Research Design and Methods

Study participants will be obese employees recruited from three employers: SEPTA, IBC, and the City of Philadelphia. We will structure the first 6 months of the study (Phase I) as a weight loss phase in which participants will be encouraged as a weight loss goal to lose 0.5 pound per week [Note: weight loss targets for sample size calculations use kg to fit in with the literature but we will use pounds for the incentive targets, as that is what Americans are most familiar with]. Phase II (months 7-18) will consist of a period of weight loss maintenance in which participants will be able to choose a weight loss goal each month of either 0 or 0.5, pounds of weight loss per week. Phase III (months 19-24) will be a period of post-intervention follow-up concluding with an in-person weigh-in at month 24.

Participants will be randomized to either a control group (information provision and the standard wellness program at each employer along with measurement of weight at 0, 6, 12, 18, and 24 months), a lottery-based financial incentive, or a series of environmental strategies and counseling regarding food intake and physical activity, or a combination of the financial incentive and environmental strategies. Participants in the incentive arm will be eligible for daily winnings based on the weights that are transmitted using wireless scales which we will provide so that they receive daily feedback on their winnings to keep weight loss goals salient. However, we will only provide the full payments based on validation of weight measurement at 6 months intervals, taking advantage of the motivating power of loss aversion by highlighting to participants that they only receive their daily winnings if they continue to maintain weight loss (or lose further weight) (an approach we have used with great success in previous studies).

8.0 **Outcome assessments**

Weight for all participants will be ascertained in-person upon enrollment into the study and then at the visits at the end of Phase I (6 months), at 12 and 18 months (end of Phase II), and at 24 months (end of Phase III). The primary outcome will be change in the in-person weight from baseline to 18 months. We will utilize other measures collected at months 6, 12, 18 and 24 as part of routine biometric assessments by the participants' employers, all of whom share the same insurance carrier, Independence Blue Cross (IBC). The allowable visit windows for these weight milestones are: -2/+4 weeks for the 6 month visit, -2/+6 weeks for the 12 and 18 month visits, and -2/+8 weeks for the 24 month visit.

9. Statistical Considerations

9.1 Analytical Plan

<u>Descriptive analyses</u>

We will produce data summaries regularly using frequencies for categorical variables and means, medians, and ranges for continuous variables. We will assess data quality and examine distributional assumptions with graphical methods. To evaluate balance among groups achieved by randomization, we will compare baseline values of all variables across the 4 arms using appropriate tests.

<u>General procedures</u>

All primary analyses will be on an intent-to-treat basis including each participant in the group to which s/he was randomized, regardless of adherence to the assigned strategy. Given the high rates of missing data typical of weight loss studies, handling of missing data is an important issue. Primary analyses will use multiple imputation for the missing in-person weight outcome at 18 months, using the randomization strata (sex, employer, initial BMI), study arm and other baseline variables as predictors in the imputation model. Baseline variables will include age, race, income, education, marital status, household size, physical activities, eating behavior index, stages of change, SF-36 General Health, and baseline weight. Sensitivity analyses will be performed with imputation models that also use post-baseline data, and recent weight loss trend before drop out, as well as an analysis that assumes that any participants for whom follow-up weight loss data are unavailable have had their weight return to baseline (weight at beginning of Pre Phase). Within each arm, this last assumption is likely to be conservative but this may not be the case in inter-arm comparisons, depending on the dropout rates in the different arms and true follow-up weights of the dropouts. Thus, a key secondary analysis will consider the effect of differential dropout among the treatment arms. For this analysis, methods that address potential patterns of MNAR may be considered in the missing data imputation, such as pattern mixture models as appropriate. Finally, we will also consider a per-protocol type analysis, which examines the difference in the intervention arms in the complete case data.

<u>Efficacy Analyses</u>

All hypotheses will be tested using two-sided, 0.05-level tests unless otherwise specified (notably the Holm testing approach will be used for the five primary hypotheses in Specific Aims 1-4). <u>The primary</u>

analyses will be an unadjusted intent-to-treat analyses, using a t-test for differences in weight change from baseline to 18 months, as measured by the in-person weight, between each intervention group and the control group, and the combined compared to each single intervention, applying the Holm-Bonferroni corrected p-value for multiple comparisons testing. If weight change appears to be non-normally distributed in the blinded data, we will find an appropriate statistical test accordingly. Missing data will be handled as described above.

Weight change between baseline and 18 months, with 95% confidence intervals (CIs), will be estimated for comparison with adjusted analyses. We will estimate regression models adjusted for the stratification variables (sex, employer, initial BMI) and other participant characteristics factors including age, race, income, and education. We will evaluate the evidence of confounding for other baseline (baseline weight, marital status, household size, physical activities- i.e total minutes of MVPA+walking/week, eating behavior index, stage of change, SF-36 General Health) using change in estimate criterion (10%). We will fit exploratory models of the repeated weight measurements that incorporate time as a polynomial function or using visit-specific indicator variables to determine the most parsimonious model that adequately describes the observed patterns, as necessary. Models will be built separately for the in-person and at home weight data. Models using the at-home weight data would be considered only as potential exploratory analyses comparing the 3 intervention arms, since at-home weights are not available for the control arm. We will investigate random-effects models that allow for baseline individual variability as well as variability in the changes in weights over time; an example is the following: $E(weight_{ij}) = \beta_0 + \beta_1 Time_j + \beta_2 Group_i + \beta_3 X_i + b_{0i} + b_{1i} Time_j$, where *i* indicates subject, *j* indicates assessment times, the parameters are fixed effects linking time, a treatment group vector, and a vector of other demographic or clinical covariates X_i to the outcomes, and b_{0i} and b_{1i} are random intercept and slope effects. Tests for significance of random effects will use likelihood ratio tests for nested models; we will compare models with different random effects structures using the maximized log-likelihoods and the Akaike Information Criterion (AIC). We will apply standard diagnostic techniques to assess model adequacy. We will use treatment by time interactions to assess whether the rate of change in weight differs by intervention arm. We hypothesize that incentives may be more effective among lower income individuals and will evaluate this using interaction terms of income with treatment group. We will explore differential effects by race and education and baseline levels of intrinsic motivation and stages of change. We will also consider other potential mediators, such as the home food and activity environment. To assess the sensitivity of treatment effect estimates to missing data, we may fit hierarchical or mixed effects models with and without accounting for informative missing data.

For Specific Aim 5, we'll consider appropriate methods for cost effective analysis such as the models proposed below. We will assess the costs of each of the intervention arms from both the employer and social perspective and compare the cost differences between each arm relative to the effectiveness measured by incremental weight loss achieved. The principal incremental cost-effectiveness ratios between intervention and control arms will be from the employer's perspective and we will compare costs during the intervention from baseline to 18 months per unit change in weight. Secondary analyses

may also 1) evaluate this same ratio but use either 6 months post- intervention data (24 month visit) or 12 months of intervention data (12 month visit) and 2) evaluate these 2 ratios using a limited social perspective. Cost models will use a generalized linear model with a log link and gamma family. Missing data strategies will parallel those described above. We will assess sampling uncertainty for the comparison of costs and effects by calculating parametric 95% CIs for the cost per kg lost and acceptability curves. Standard errors and correlation of the difference in cost and effect will be derived using a bootstrap procedure.

9.2 Power and Sample Size Considerations

We have designed the study with adequate power to detect differences in weight loss over an 18-month period. Our intervention should achieve its maximal impact in maintaining initial weight loss at the end of Phase II (month 18), when the incentive payments cease. To maintain the experiment-wide Type I error and guard against false conclusions of effectiveness, we will use the Holm multiple comparisons adjustment for test the 5 primary comparisons in Specific Aims 1-4 . If the interventions are as effective as hypothesized, the proposed sample size maintains greater than 90% power to show significance for each of the three intervention arms compared to control and greater than 80% power to show significance for the combined intervention group compared to each of the two interventions alone.

It is important to note that while we originally planned to recruit 328 participants, a small subset was unable to successfully set up their Withings scales post randomization due to Wi-Fi connectivity issues. Therefore, we increased our target sample size by the number of people who were unable to connect their Withings scales to the Way To Health platform (16 additional subjects), bringing our recruitment total to 344 participants. The final statistical analysis is adjusted for the stratification factors to account for any imbalances that may have occurred. We have previously communicated this information to the IRB and the members of the Data Safety and Monitoring Board (DSMB), who approved this approach. The following information outlines our original power calculations and sample size considerations.

We plan to recruit approximately one third of the participants required for the start of Phase I from each of our participating employers (IBC, SEPTA, and City of Philadelphia). Given the large number of potentially eligible employees, we can, if needed, easily increase the proportion of participants from any of these employers to meet recruitment targets. Participants will be randomized to our 3 intervention groups and the control group using a 1:1 ratio for the intervention arms. We built in a margin of 20% of our original target sample size of 328 for potential attrition before the 18-month assessment, resulting in an expected 260 participants (65 per arm) who are available for analysis at the end of Phase II. This sample size will provide us with greater than 90% power to detect a difference in weight change between baseline and the 18-month weigh-in of 5 kg between each single intervention group and the control group (primary outcomes for Specific Aims 1-3) and 87% power to detect a 3 kg difference between the combined group and either the incentive or environmental strategies groups (Specific Aim 4). Namely, we will have greater than 85% power for the 5 primary comparisons of interest for an expected weight change between months 0-18 of -8 kg, -5 kg and 0 kg in the combined, intervention and control groups, respectively (a net difference between single intervention and control groups of 5 kg,

combined intervention and control groups of 8 kg, and 3kg between the single interventions and combined group) and assuming a standard deviation (S.D.) of weight loss of 5 kg. We will also have greater than 80% power to detect a difference of 4.6 kg in weight change between single intervention and control groups and 7.3 kg between the combined and control groups (primary outcomes for Specific Aims 1-3), while maintaining approximately 80% power for a 2.7 kg difference between the combined and each single intervention (Specific Aim 4). For our secondary outcomes (Specific Aim 5), we wish to compare the cost differences between each arm relative to the effectiveness measured by incremental weight loss achieved. We will calculate a point estimate of cost per kg of weight loss based on estimated inter-arm differences in weight loss and the incremental cost of the interventions, as described in C.3.d.ii.c. For weight loss at 18 months, given an expected incremental cost of $810 (incentives) and incremental weight loss of 5 kg, 328 participants provide 80% power to detect a cost per kg lost of $430 and greater than 90% power to detect a cost per kg lost of $575. This study is primarily a test of the efficacy of these interventions. Due to resource constraints and because we do not yet know about intervention effectiveness, we did not power this study based on cost effectiveness analyses. Estimated detectable costs per kg are a function of wide confidence intervals due to sample size.

10.0 Measurement of effect:

The goal of this analysis will be to assess relative weight loss in the intervention arms relative to control. For the principal cost-effectiveness analysis, measurement of incremental weight loss will be based on differences in weight between baseline and the 18 month visit; in secondary analyses it will be based on weight differences between baseline and 24 months and baseline and 12 months.
