# Supplementary material for: Effect of Financial Incentives and Environmental Strategies on Weight Loss in the Healthy Weigh Study: A Randomized Clinical Trial
Source: JAMA Netw Open. 2021 Sep 7;4(9):e2124132. doi: 10.1001/jamanetworkopen.2021.24132 (PMC8424479; doi:10.1001/jamanetworkopen.2021.24132)
Supplement: Supplement 2. — eTable 1. Analysis of the Main Effect for Weight Change (lbs.) at 18 and 24 Months in the Complete Case Population eTable 2. Unadjusted Analysis of Exploratory Outcomes and Potential Mechanisms of Change Between Baseline and 18 Months Among Complete Case and Intention-to-Treat Populations eTable 3. Analysis of the Main Effect for Weight Change (lbs.) at 18 Months in Per-Protocol Analysis Population eTable 4. Analysis of Frequency of In-Home Weigh-In per Week by Intervention Phase (N=244) eFigure 1. Mean of Weight Change (lbs.) From Baseline to Different Time Points by Arm in Intention-to-Treat Population (N=344) eFigure 2. Mean of Weight Change (lbs.) From Baseline at Different Time Points by Arm in Complete Case Population [file jamanetwopen-e2124132-s002.pdf]

# Supplemental Online Content

Glanz K, Shaw PA, Kwong PL, et al. Effect of financial incentives and environmental strategies on weight loss in the Healthy Weigh Study: a randomized clinical trial. *JAMA Netw Open*. 2021;4(9):e2124132. doi:10.1001/jamanetworkopen.2021.24132

**eTable 1.** Analysis of the Main Effect for Weight Change (lbs.) at 18 and 24 Months in the Complete Case Population
**eTable 2.** Unadjusted Analysis of Exploratory Outcomes and Potential Mechanisms of Change Between Baseline and 18 Months Among Complete Case and Intention-to-Treat Populations
**eTable 3.** Analysis of the Main Effect for Weight Change (lbs.) at 18 Months in Per-Protocol Analysis Population
**eTable 4.** Analysis of Frequency of In-Home Weigh-In per Week by Intervention Phase (N=244)
**eFigure 1.** Mean of Weight Change (lbs.) From Baseline to Different Time Points by Arm in Intention-to-Treat Population (N=344)
**eFigure 2.** Mean of Weight Change (lbs.) From Baseline at Different Time Points by Arm in Complete Case Population

This supplemental material has been provided by the authors to give readers additional information about their work.

© 2021 Glanz K et al. *JAMA Network Open.*

**eTable 1. Analysis of the Main Effect for Weight Change (lbs.) at 18 and 24 Months in the Complete Case Population**

| Comparisons | Unadjusted analysis (N=228) | | Adjusted analysis (N=220) | |
|---|---|---|---|---|
| | Effect size (95% CI) | P Value | Effect size (95% CI) | P Value |
| **From baseline to 18 months** | | | | |
| Incentive vs. Usual Care | -6.6 (-12.6, -0.6) | 0.03 | -6.6 (-12.5, -0.8) | 0.03 |
| ES. vs. Usual Care | -3.9 (-9.6, 1.8) | 0.18 | -3.4 (-9.0, 2.2) | 0.23 |
| Combined vs. Usual Care | -2.9 (-8.2, 2.4) | 0.29 | -2.8 (-8.7, 3.1) | 0.35 |
| Incentive vs. Combined | -3.7 (-10.0, 2.6) | 0.24 | -3.8 (-10.0, 2.4) | 0.23 |
| ES. vs. Combined | -1.0 (-7.1, 5.0) | 0.74 | -0.6 (-6.6, 5.4) | 0.86 |
| Incentive + Combined vs. ES. + Usual Care | -2.7 (-6.9, 1.5) | 0.21 | -3.1 (-7.4, 1.1) | 0.15 |
| ES. + Combined vs. Incentive + Usual Care | -0.4 (-4.6, 3.9) | 0.87 | -0.2 (-4.4, 4.0) | 0.91 |
| **From baseline to 24 months** | | | | |
| Incentive vs. Usual Care | -4.8 (-11.9, 2.4) | 0.19 | -5.6 (-11.9, 0.7) | 0.08 |
| ES. vs. Usual Care | -4.8 (-10.6, 1.1) | 0.11 | -4.9 (-11.0, 1.2) | 0.11 |
| Combined vs. Usual Care | -3.8 (-9.2, 1.6) | 0.16 | -5.4 (-11.7, 0.8) | 0.09 |
| Incentive vs. Combined | -1.0 (-8.2, 6.3) | 0.79 | -0.2 (-6.7, 6.3) | 0.96 |
| ES. vs. Combined | -1.0 (-7.0, 5.0) | 0.75 | 0.5 (-5.7, 6.7) | 0.87 |
| Incentive + Combined vs. ES. + Usual Care | -1.9 (-6.5, 2.8) | 0.43 | -3.1 (-7.5, 1.4) | 0.18 |
| ES. + Combined vs. Incentive + Usual Care | -2.1 (-6.7, 2.5) | 0.37 | -2.6 (-7.1, 1.9) | 0.26 |
| **From 18 months to 24 months** | | | | |
| Incentive vs. Usual Care | 1.8 (-2.4, 6.0) | 0.40 | 3.2 (-0.9, 7.3) | 0.13 |
| ES. vs. Usual Care | -0.9 (-4.2, 2.4) | 0.59 | -0.6 (-4.5, 3.2) | 0.75 |
| Combined vs. Usual Care | -1.0 (-4.3, 2.4) | 0.57 | -1.1 (-5.2, 3.0) | 0.59 |
| Incentive vs. Combined | 2.7 (-2.0, 7.4) | 0.25 | 4.3 (0, 8.6) | 0.05 |
| ES. vs. Combined | 0.1 (-3.9, 4.0) | 0.98 | 0.5 (-3.7, 4.6) | 0.83 |
| Incentive + Combined vs. ES. + Usual Care | 0.8 (-2.0, 3.7) | 0.56 | 1.3 (-1.7, 4.2) | 0.40 |
| ES. + Combined vs. Incentive + Usual Care | -1.7 (-4.5, 1.0) | 0.22 | -2.2 (-5.1, 0.7) | 0.14 |

Generalized linear models are adjusted by the randomization strata variables of sex, employer and initial BMI, study arm, and baseline participant characteristics of age, race, annual household income, education, baseline weight, marital status, household size and stage of change.

© 2021 Glanz K et al. *JAMA Network Open.*

**eTable 2. Unadjusted Analysis of Exploratory Outcomes and Potential Mechanisms of Change Between Baseline and 18 Months Among Complete Case and Intention-to-Treat Populations**

| Comparisons | Unadjusted (complete case, N=269) | | Unadjusted (ITT, N=344) | |
|---|---|---|---|---|
| | Effect size (95% CI) | p value | Effect size (95% CI) | p value |
| **Total physical activity[@] (minute)** | | | | |
| Incentive vs. Usual Care | -93.6 (-483.2, 296.0) | 0.63 | -18.9 (-405.2, 367.5) | 0.92 |
| ES vs. Usual Care | -57.8 (-434.6, 319.1) | 0.76 | -83.6 (-465.7, 298.6) | 0.67 |
| Combined vs. Usual Care | -235.6 (-548.5, 77.3) | 0.14 | 3.8 (-350.7, 358.2) | 0.98 |
| Incentive vs. Combined | 142.0 (-262.2, 546.2) | 0.49 | -22.6 (-413.7, 368.4) | 0.91 |
| ES vs. Combined | 177.8 (-214.2, 569.8) | 0.37 | -87.3 (-485.9, 311.3) | 0.67 |
| Incentive vs. Non-incentive | -123.6 (-408.2, 160.9) | 0.39 | 34.2 (-245.3, 313.8) | 0.81 |
| ES vs. Non-ES | -84.7 (-368.5, 199.0) | 0.56 | -30.5 (-301.7, 240.7) | 0.83 |
| **Cognitive restraint scale[#] (0 to 100)** | | | | |
| Incentive vs. Usual Care | 4.7 (-2.0, 11.3) | 0.17 | 5.7 (-1.0, 12.4) | 0.10 |
| ES vs. Usual Care | 8.9 (2.9, 15.0) | 0.004 | 10.0 (3.8, 16.2) | 0.002 |
| Combined vs. Usual Care | 2.2 (-4.1, 8.5) | 0.49 | 3.6 (-2.9, 10.0) | 0.28 |
| Incentive vs. Combined | 2.4 (-4.6, 9.5) | 0.49 | 2.1 (-4.9, 9.1) | 0.56 |
| ES vs. Combined | 6.7 (0.2, 13.2) | 0.04 | 6.4 (-0.3, 13.1) | 0.06 |
| Incentive vs. Non-incentive | -1.0 (-5.7, 3.7) | 0.67 | -0.4 (-5.2, 4.4) | 0.88 |
| ES vs. Non-ES | 3.7 (-1.0, 8.3) | 0.12 | 3.9 (-0.7, 8.6) | 0.10 |
| **Uncontrolled eating scale[#] (0 to 100)** | | | | |
| Incentive vs. Usual Care | -1.5 (-6.6, 3.6) | 0.56 | -0.3 (-5.5, 4.8) | 0.91 |
| ES vs. Usual Care | -1.7 (-6.1, 2.7) | 0.45 | -0.4 (-5.0, 4.1) | 0.85 |
| Combined vs. Usual Care | 2.3 (-2.8, 7.3) | 0.38 | 3.6 (-1.4, 8.6) | 0.16 |
| Incentive vs. Combined | -3.8 (-9.4, 1.9) | 0.19 | -3.9 (-9.2, 1.4) | 0.15 |
| ES vs. Combined | -3.9 (-9.0, 1.1) | 0.13 | -4.0 (-8.9, 0.8) | 0.10 |
| Incentive vs. Non-incentive | 1.1 (-2.5, 4.7) | 0.54 | 1.9 (-1.7, 5.5) | 0.31 |
| ES vs. Non-ES | 0.8 (-2.7, 4.3) | 0.66 | 1.7 (-1.8, 5.2) | 0.34 |
| **Emotional eating scale[#] (0 to 100)** | | | | |
| Incentive vs. Usual Care | -1.8 (-8.9, 5.3) | 0.62 | -1.0 (-8.2, 6.3) | 0.80 |
| ES vs. Usual Care | 0.5 (-5.8, 6.9) | 0.87 | 0.6 (-6.0, 7.2) | 0.85 |
| Combined vs. Usual Care | -0.5 (-8.3, 7.4) | 0.90 | -0.4 (-7.9, 7.2) | 0.92 |
| Incentive vs. Combined | -1.3 (-9.5, 6.9) | 0.76 | -0.6 (-8.2, 7.0) | 0.88 |
| ES vs. Combined | 1.0 (-6.6, 8.6) | 0.79 | 1.0 (-6.3, 8.3) | 0.79 |
| Incentive vs. Non-incentive | -1.4 (-6.6, 3.7) | 0.58 | -1.0 (-6.2, 4.3) | 0.71 |
| ES vs. Non-ES | 0.9 (-4.1, 6.0) | 0.72 | 0.6 (-4.4, 5.6) | 0.81 |
| **SF-36 general health[*] (1 to 100)** | | | | |
| Incentive vs. Usual Care | 0.4 (-4.9, 5.7) | 0.89 | -1.0 (-6.2, 4.3) | 0.72 |
| ES vs. Usual Care | 7.0 (2.0, 11.9) | 0.006 | 5.6 (0.7, 10.6) | 0.03 |
| Combined vs. Usual Care | 4.8 (-0.5, 10.2) | 0.08 | 4.5 (-0.9, 9.8) | 0.10 |
| Incentive vs. Combined | -4.5 (-10.1, 1.1) | 0.12 | -5.4 (-11.0, 0.1) | 0.06 |
| ES vs. Combined | 2.1 (-3.1, 7.4) | 0.42 | 1.2 (-4.0, 6.4) | 0.66 |
| Incentive vs. Non-incentive | -1.1 (-4.8, 2.7) | 0.58 | -1.1 (-4.8, 2.7) | 0.57 |
| ES vs. Non-ES | 5.8 (2.2, 9.5) | 0.002 | 5.5 (1.8, 9.3) | 0.004 |

© 2021 Glanz K et al. *JAMA Network Open.*

[@] Measured using the International Physical Activity Questionnaire.
[#] Eating behavior control is measured by Three Factor Eating Questionnaire.
The raw eating scale scores are transformed to a 0–100 scale [((raw score – lowest possible raw score)/possible raw score range) × 100].
Higher scores in the respective scales are indicative of greater cognitive restraint, uncontrolled, or emotional eating.
[*] General health is assessed using SF-36 with default range from 1 to 100. Higher value means better health condition.

© 2021 Glanz K et al. *JAMA Network Open.*

**eTable 3. Analysis of the Main Effect for Weight Change (lbs.) at 18 Months in Per-Protocol Analysis Population***

| Comparisons | Unadjusted analysis (N=209) | | Adjusted analysis** (N=188) | |
|---|---|---|---|---|
| | Effect size (95% CI) | P Value | Effect size (95% CI) | P Value |
| **From baseline to 18-month** | | | | |
| Incentive vs. Usual Care | -8.3 (-14.9, -1.7) | 0.02 | -9.1 (-15.7, -2.5) | 0.007 |
| ES. vs. Usual Care | -4.4 (-10.4, 1.7) | 0.16 | -5.7 (-12.1, 0.7) | 0.08 |
| Combined vs. Usual Care | -4.6 (-10.4, 1.2) | 0.12 | -3.8 (-10.4, 2.8) | 0.26 |
| Incentive vs. Combined | -3.7 (-11, 3.6) | 0.31 | -5.3 (-12.5, 1.9) | 0.15 |
| ES. vs. Combined | 0.2 (-6.6, 7) | 0.95 | -1.9 (-8.8, 4.9) | 0.58 |
| Incentive + Combined vs. ES. + Usual Care | -4.4 (-9.1, 0.3) | 0.07 | -3.9 (-8.7, 1) | 0.12 |
| ES. + Combined vs. Incentive + Usual Care | -0.9 (-5.5, 3.8) | 0.71 | -1.1 (-6, 3.8) | 0.65 |

*Per protocol analysis population inclusion criteria:

1. Incentive arm need to have in-home weigh-in at least 1 time per week on average;
2. Environmental strategies (ES) arm need to use 10 or more tips;
3. Combined arm need to have in-home weigh-in at least 1 time per week on average and use 10 or more tips;
4. All four arms need to attend 6-, 12-, and 18-month in-site weigh-in visits

**Generalized linear models are adjusted by the randomization strata variables of sex, employer and initial BMI, study arm, and baseline participant characteristics of age, race, annual household income, education, baseline weight, marital status, household size and stage of change.

© 2021 Glanz K et al. *JAMA Network Open.*

**eTable 4. Analysis of Frequency of In-Home Weigh-In per Week by Intervention Phase (N=244).**

| Variable | Intervention ( week 2 to week 72) | | Post-intervention (week 75 to week 103) | |
|---|---|---|---|---|
| | Effect size (95% CI) | P-Value | Effect size (95% CI) | P-Value |
| **Model with only arm effect (ref: ES.)** | | | | |
| Combined | 1.4 (0.8, 2.0) | <.001 | 0.3 (-0.3, 0.8) | 0.35 |
| Incentive | 1.6 (1.0, 2.3) | <.001 | 0.3 (-0.3, 1.0) | 0.29 |
| **Model with arm (ref: ES.) and week interaction** | | | | |
| Combined | 1.5 (0.9, 2.1) | <.001 | 1.8 (-0.5, 4.1) | 0.13 |
| Incentive | 1.8 (1.1, 2.4) | <.001 | 1.9 (-0.5, 4.3) | 0.11 |
| Week | -0.02 (-0.03, -0.01) | <.001 | -0.03 (-0.04, -0.01) | <.001 |
| Combined*week | -0.003 (-0.015, 0.009) | 0.59 | -0.017 (-0.039, 0.006) | 0.14 |
| Incentive*week | -0.004 (-0.015, 0.007) | 0.47 | -0.018 (-0.041, 0.006) | 0.14 |

Generalized linear models are tested the effect of frequency of at-home weigh-in per week at different intervention phase. Subjects who did not have scales are excluded in the analysis, and who withdrew are excluded from the denominator from the week they withdrew. The first week data is not included in the intervention phase as it is a grace period for setting up scales, and week 73 and 74 data are excluded in post-intervention phase as it is a wash-out period.

© 2021 Glanz K et al. *JAMA Network Open.*

**eFigure 1. Mean of Weight Change (lbs.) From Baseline to Different Time Points by Arm in Intention-to-Treat Population (N=344).**

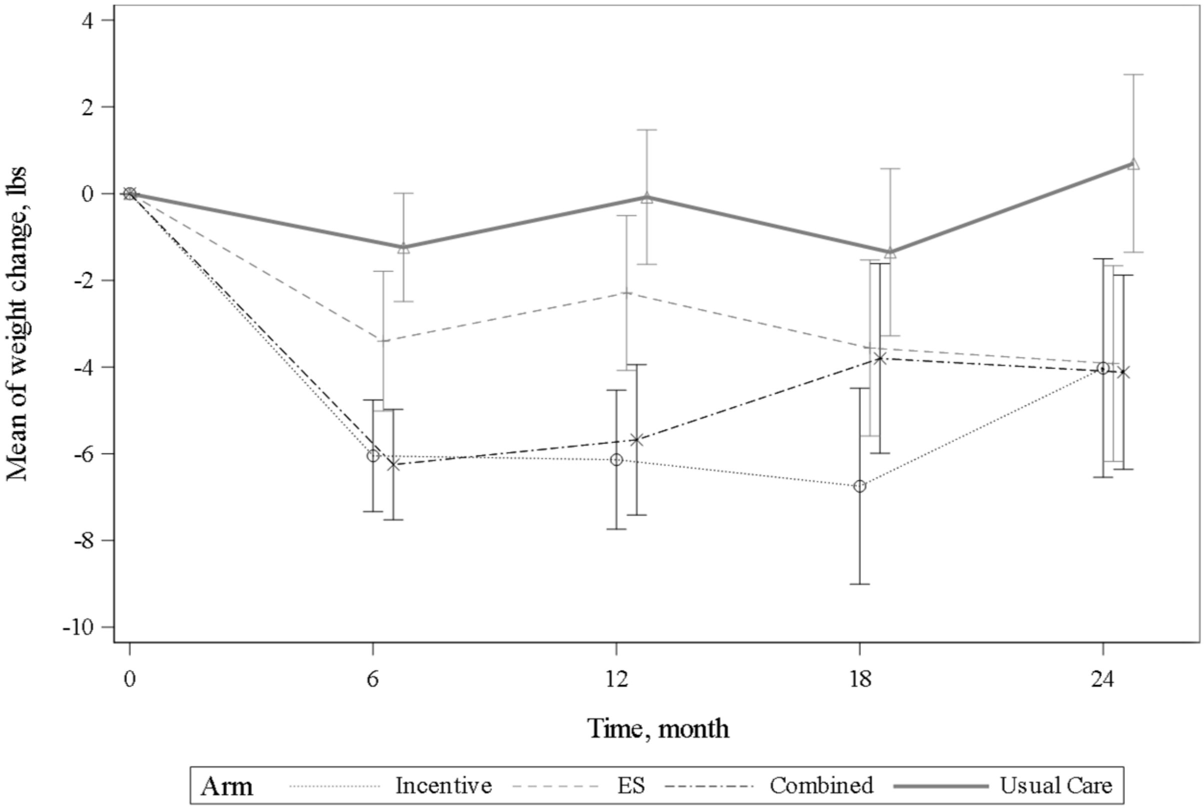

**eFigure 2. Mean of Weight Change (lbs.) From Baseline at Different Time Points by Arm in Complete Case Population**

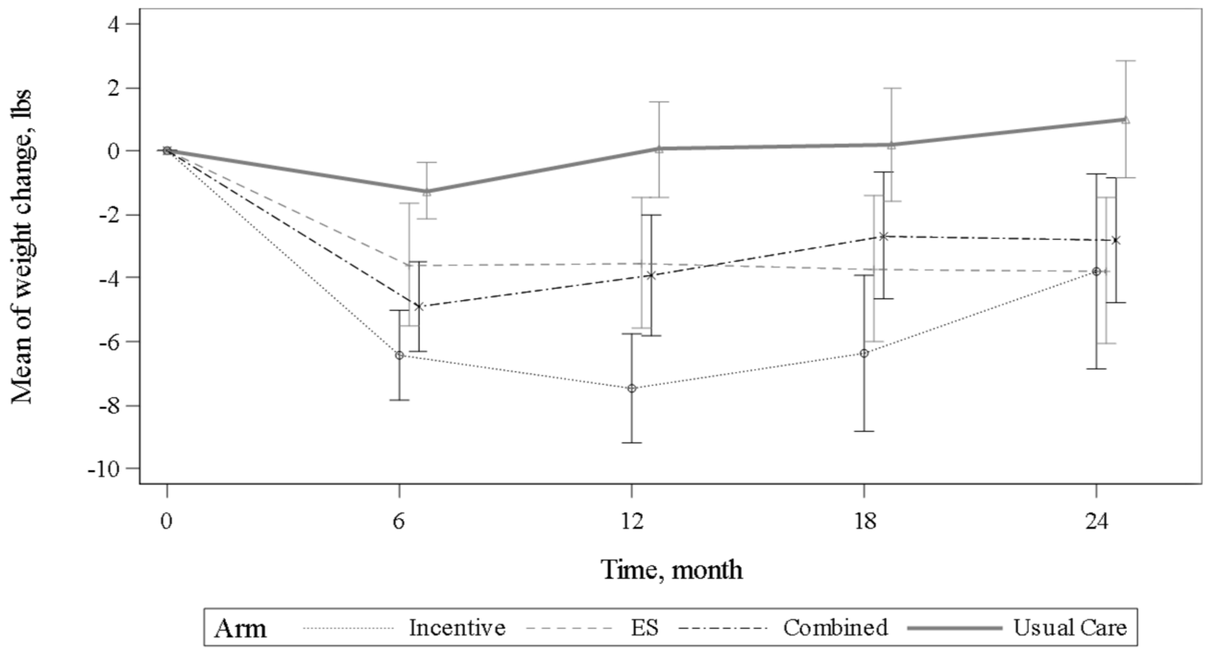


*Number of participants:*

| | | | | | |
|---|---|---|---|---|---|
| Incentive | 52 | 49 | 50 | 52 | 52 |
| ES | 62 | 59 | 59 | 62 | 62 |
| Combined | 54 | 49 | 52 | 54 | 54 |
| Usual Care | 60 | 55 | 60 | 60 | 60 |

© 2021 Glanz K et al. *JAMA Network Open.*
