# Supplementary material for: Effect of Financial Incentives and Environmental Strategies on Weight Loss in the Healthy Weigh Study: A Randomized Clinical Trial
Source: JAMA Netw Open. 2021 Sep 7;4(9):e2124132. doi: 10.1001/jamanetworkopen.2021.24132 (PMC8424479; doi:10.1001/jamanetworkopen.2021.24132)
Supplement: Supplement 3. — Data Sharing Statement [file jamanetwopen-e2124132-s003.pdf]

Data Sharing Statement

Glanz. Effect of Financial Incentives and Environmental Strategies on Weight Loss in The Healthy Weigh Study. *JAMA Netw Open*. Published September 07, 2021. doi:10.1001/jamanetworkopen.2021.24132

Data
Data available: Yes
Data types: Deidentified participant data
How to access data: kglanz@upenn.edu
When available: With publication

Supporting Documents
Document types: None

Additional Information
Who can access the data: Researchers whose proposed use of the data has been approved.
Types of analyses: For a specified purpose.
Mechanisms of data availability: With a signed data access agreement.
